# Supplementary material for: The January 2022 Hunga eruption cooled the southern hemisphere in 2022 and 2023
Source: Commun Earth Environ. 2025 Mar 27;6(1):240. doi: 10.1038/s43247-025-02181-9 (PMC11949836; doi:10.1038/s43247-025-02181-9)
Supplement: Supplementary file 2 — Supplementary Information [file 43247_2025_2181_MOESM2_ESM.pdf]

Supplementary Information

The January 2022 Hunga eruption cooled the southern hemisphere in 2022 and 2023

Ashok Kumar Gupta[1*], Tushar Mittal[2], Kristen E. Fauria[3], Ralf Bennartz[3], and Jasper F. Kok[1]

[1]Department of Atmospheric and Oceanic Sciences, University of California, Los Angeles, CA, 90095, USA

[2]Department of Geosciences, Pennsylvania State University, University Park, PA, USA

[3]Department of Earth and Environmental Sciences, Vanderbilt University, Nashville, TN, USA

**Corresponding author (& present Address)*: Ashok Kumar Gupta, ashokgupta@atmos.ucla.edu

This PDF file includes:

Supplementary Note

Supplementary Results

Supplementary Methods

Supplementary Figures 1-19

Supplementary Table 1

Supplementary References

Supplementary Note

Previous observational work on Hunga eruption's radiative forcing

Recent studies on the climate impacts of the Hunga eruption have not considered the combined radiative effects of perturbed stratospheric water vapor, aerosols, and ozone using a radiative transfer model during the two years following the 2022 eruption. Thus, the conclusion that Hunga warmed the planet—and the associated dynamical and climatic changes—based solely on its water vapor input may be premature[1]. Previous studies[2], which examined the initial two months of water vapor profiles after the eruption, indicated that the Hunga eruption was likely to warm the planet during the sulfate aerosol growth phase. By sulfate aerosol growth phase, we refer to the period during which volcanic sulfur dioxide is converted into sulfate aerosols via nucleation, coagulation, and hygroscopic growth[3]. Stratospheric sulfate aerosols from large volcanic eruptions can remain in the stratosphere for months to a few years, gradually dispersing and settling out depending on factors such as injection height, particle size, coagulation, nucleation, and stratospheric circulation[1,3]. Duchamp[4] also concluded that the Hunga eruption would warm the planet based on water vapor.

To our knowledge, only few studies[5,6,7] have suggested that the Hunga eruption could cool the planet. Zhu[6] used global climate models to simulate the effect of enhanced SH_2O on SO_2 lifetime and aerosol evolution, finding that water vapor leads to more efficient aerosol growth, resulting in larger extinction and a global radiative flux of approximately –0.1 to –0.2 W m^{-2} during the two months following the eruption. Schoeberl[5] estimated a net radiative forcing based on a combination of scaling analyses from past subaerial eruptions (for aerosols) and radiative calculations for water vapor, while Schoeberl[7] used similar scaling methods to demonstrate a reduction in the net downward radiative flux at the tropopause due to water vapor, aerosols, and ozone during the first two years after the eruption. Thus, none of the existing studies has explicitly accounted for the impact of stratospheric aerosols using multi-wavelength aerosol extinction-based radiative properties constrained by observations from 2022 and 2023 post-Hunga eruption. Zhu[6] modeling results underscore that this variable is important for determining the net radiative forcing.

Following our analysis using the 2D-filtered technique (see Methods) for ozone species based on Wilmouth[8] and Santee[9], we demonstrate that seasonal changes in ozone concentration following the Hunga eruption exhibit a clear signal relative to background variations (Fig. 2c and Supplementary Figures 9-14). Previous studies have also shown that ozone concentration responded to the Hunga eruption[9-13]. Incorporating the 2D-filtered ozone changes into our calculations of the Hunga eruption's radiative response clarifies how the eruption influences both the sign and magnitude of this response. Our findings reveal that in 2022, ozone loss in the Southern Hemisphere further reduced the combined TOA radiative forcing while increasing the near-tropopause radiative forcing associated with aerosol and water vapor. In contrast, in 2023, ozone gains in SH influenced the combined radiative forcing of aerosol and water vapor.

Effect of Hunga Tonga volcanic eruption on Ozone and the TOA radiative forcing

Changes in stratospheric ozone and their radiative effects

Based on the 2D-filtered technique applied to SAGE-III/ISS ozone observations, we show a seasonal decrease in ozone concentration of approximately 1–2 DU (Fig. 2c) during 2022. In contrast, the unfiltered technique indicates a decrease of around 5–10 DU during the same period (Fig. 2d), particularly in the Southern East Pacific and Indian Ocean near the eruption site. Seasonal analysis of ozone changes from 2017 to 2021 reveals that the influence of the 2022 Hunga eruption is anomalous when assessed using the 2D-filtered technique (Supplementary Fig. 9). This finding is further supported by nearly 16 years of long-term ozone data from Aura MLS (Supplementary Fig. 13 for 2D-filtered results with water vapor). Based on the 2D-filtered data from both SAGE-III and MLS, we observe a clear ozone loss in 2022 and an ozone gain in 2023. However, disentangling the influence of different physical processes—such as chemical versus meteorological factors—in the observed ozone loss and gain requires detailed atmospheric modeling equipped with stratospheric chemistry[15-18], which is beyond the scope of this study.

Perturbations in stratospheric ozone affect Earth's radiative budget by absorbing radiation in both the infrared (longwave) and ultraviolet (shortwave)[19] regions. When the stratospheric ozone layer is depleted, less solar radiation is absorbed, allowing more radiation to be reflected

or upwelled. This results in a shortwave cooling effect at TOA, which contrasts with the positive shortwave radiative forcing observed near the tropopause. Additionally, the longwave radiative forcing is negative at both the TOA and the tropopause, further enhancing the cooling effect. These immediate radiative responses to ozone depletion are consistent with the findings of Shine[20] (see Table 5 therein). Overall, the net radiative cooling effect of reduced ozone concentration in the Southern Hemisphere may counteract, or at least influence, the warming driven by increased water vapor.

Location of stratospheric water vapor layer and aerosol layer after the Hunga eruption

Even one year after the eruption, the SH_2O layer has not descended relative to the stratospheric aerosol (Fig. 1a, b). Several factors could contribute to this, including the influence of the tropical Brewer-Dobson circulation, reduced vertical mixing in the stratosphere compared to the troposphere, and the presence of a temperature inversion in the stratosphere. It is important to note that determining the exact cause of the static injection height of the SH_2O layer is beyond the scope of this study. In the second year, however, the SH_2O layer ascends further into the upper stratosphere and spreads across both hemispheres.

For an explosive eruption, the initial injection height of the sulfur gas plume—and the corresponding altitude of its oxidation product, SO_2—determines the subsequent horizontal and vertical evolution of the sulfuric acid aerosol particles[23–25]. Similarly, for the Hunga eruption, the intensity and spatial extent of aerosol loading, along with the resulting radiative heating rates, could vertically uplift the aerosol layer. This uplift may influence both the aerosol lifetime and stratospheric temperatures, as indicated by observations following the Pinatubo eruption[24], and represents an important parameter for further quantification.

Radiative kernels of water vapor and ozone instantaneous radiative forcing at three levels: TOA, 16 km and 11 km

We estimated the radiative kernels for the instantaneous infrared radiative forcing of water vapor and ozone at three levels—the TOA, 16 km, and 11 km—for a perturbation of +1 ppmv per km (Supplementary Figures. 15-16). For water vapor, our results show that the instantaneous infrared radiative forcing is positive at 16 km and 11 km, but negative at the TOA

(Supplementary Figure 15). These estimates are consistent with those reported by Solomon[26] and Wang[27], with our sign conventions now aligned with their findings.

For ozone, a +1 ppmv per km perturbation yields a positive instantaneous radiative forcing at all three levels (TOA, 16 km, and 11 km; Supplementary Figure 16). Additionally, we find that the changes in instantaneous infrared radiative forcing due to a temperature increase of +1 K per km, a water vapor increase of +1 ppmv per km, and the combined effects of both perturbations are additive under idealized conditions (Supplementary Figure 17). Similarly, we computed these three perturbations—temperature only, ozone only, and the combined temperature and ozone effects—for both instantaneous longwave (LW; Supplementary Figure 18) and shortwave (SW; Supplementary Figure 19) radiative forcing.

Supplementary Methods

Observational data analysis approach

We estimated the increase in the SH_2O by taking vertical integral of the water-vapor content over a given altitude (z) range from lower stratosphere to upper stratosphere to compute the near global seasonal distribution of water vapor content in kg m^{-2} using SAGE-III/ISS[28] The equation for this analysis is:

$$SWV_{CL}[\theta_k, \phi_j] = \frac{1}{\rho_w} \sum_{z_i=20km}^{z_i=42km} q_v(\theta_k, \phi_j, z_i)\rho_a(\theta_k, \phi_j, z_i)dz_i \tag{1}$$

where $q_v(\theta_k, \phi_j, z_i)$ indicates the 3-D distribution of stratospheric specific humidity [g kg^{-1}], $\rho_a(\theta, \phi, z_i)$ is the 3-D distribution of density of air [kg m^{-3}], and $\rho_w = 1000$ [kg m^{-3}] is the standard density of water [kg m^{-3}].

We also estimated the zonal-mean latitude-altitude distribution of water vapor as follows:

$$SWV_{ZM}[\theta_k, z_i] = \frac{\sum_{\phi_j} c_{H_2O}[\theta_k, \phi_j, z_i]}{\sum_{\phi_j} n_j(\theta_k, \phi_j)} \tag{2}$$

where $\sum_{\phi_j} c_{H_2O}[\theta_k, \phi_j, z_i]$ is the zonally-summed H_2O concentration [cm^{-3}] from SAGE-III/ISS, and $\sum_{\phi_j} n_j(\theta_k, \phi_j)$ is total counts of zonally-summed values.

We used SAGE-III observed ozone profile in [cm^{-3}] to determine the ozone molecules per unit area by multiplying with the vertical height bin (fixed to 0.5 km) of the stratospheric layer from lower stratosphere to upper stratosphere. It is estimated as:

$$O_{3_{CL}}[\theta_k, \phi_j] = \sum_{z_i=lower\ strato}^{z_i=upper\ strato} c_{O_3}(\theta_k, \phi_j, z_i)dz_i \tag{3}$$

with the final O_3 values converted into DU units (1 DU = 2.0823 x 10^{20} molecules-m^{-2}).

Analogous to water vapor, we used an equation similar to Eq. (3) for estimating the zonal-mean of the ozone concentration and approximatively 521 nm aerosol extinction coefficient.

We integrated the aerosol extinction coefficient from lower stratosphere to upper stratosphere altitude layer to obtain the stratospheric aerosol optical depth (SAOD):

$$\mathrm{SAOD}\,[\theta_k, \phi_j] = \sum_{z_i=\text{lower strato}}^{z_i=\text{upper strato}} \beta_{ext}(\theta_k, \phi_j, z_i) \times dz_i \qquad (4)$$

Here, β_{ext} [km $^{-1}$] is the aerosol extinction coefficient and subscript dz_i runs over the bins of 0.5 km. $\mathrm{SAOD}_{mean} = \frac{\Sigma_{\phi_j} \mathrm{SAOD}\cos(\phi_j)}{\Sigma_{\phi_j}\cos(\phi_j)}$ denotes the near-global mean SAOD.

Idealized model set up: LibRadtran Radiative Transfer model

We utilized the LibRadtran (library for radiative transfer calculations[30-31]) model to simulate the 3-D distribution of downward and upward irradiances and atmospheric radiative heating rates in different spectral ranges: SW (0.28–4.0 µm) and LW (4.0–100µm). For this radiative computation, the REPTRAN (Representative Wavelengths Parameterization Approach[32]) was employed for radiative features of spectrally-integrated quantities such as water vapor and ozone. We computed the profiles of fluxes and heating rates using the discrete ordinate solver (DISORT[33]) with a total 8 streams.

For both the Hunga-2022 and Hunga-2023 eruption periods, the basic background 5-year mean meteorological profiles, such as temperature profiles, remain unchanged. Consequently, the radiative forcing calculations primarily involve instantaneous radiative forcing calculations. The only perturbations made are to the three radiatively significant stratospheric species: stratospheric water vapor, stratospheric sulfate aerosol, and ozone.

Background mean climatology (2017-2022) of mid-latitudes mean clear-sky surface albedo has seasonal values 0.14, 0.12, 0.11, and 0.12, respectively, during northern winter, autumn summer and fall. Thus, in this idealized radiative transfer simulation, the surface albedo was assumed to be 0.15 between the latitudinal band of 60°N-60°S for both the background (CLIM) and Hunga-2022 (and Hunga-2023) periods. By assuming the same albedo value for both the background and Hunga-2022 (and Hunga-2023) periods, the simulation assumes that any difference in radiative transfer calculations between these periods (Hunga versus CLIM) is not influenced by cloudy/clear-sky surface reflectivity changes.

In our idealized study, fixing the clear-sky surface albedo is valuable for gaining insights into specific radiative impacts of volcanic sulfate aerosol and isolating the surface albedo effects related to changing surface characteristics, dynamical and cloud properties, and radiative feedback effects. We note that this assumption of fixed clear-sky surface albedo will not drastically influence the sign of net radiative cooling of Hunga eruption as cooling effect of volcanic eruptions on the simplified homogenous surface are well understood[34].

Seasonal-mean daily-insolation weighted solar zenith angle values were used to account for fluctuations in solar radiation intensity throughout the season. This approach considered the varying intensity of solar radiation at different times of the year, rather than relying on solar zenith angle in each season[35]. We used SAGE-III/ISS observed temperature profiles above 9 km and ERA5 reanalysis temperature profiles below 9 km. We note that using the diurnally-averaged temperature profile is important for accurate radiative transfer simulations[36]. However, due to the lack of day-night average profiles of three radiative species from SAGE III which makes measurements only in daytime (solar data is much higher signal-to-noise ratio compared to lunar), we used only the daytime temperature profiles for this idealized setup of radiative transfer simulations.

Our primary focus in this paper is evaluating the instantaneous radiative response at the TOA and near the tropopause without including any temperature adjustments in the stratosphere or troposphere.

Quality of SAGE-III/ISS observations of three stratospheric species

For a given species, we used only those profiles at a given altitude when there was confidence in the retrieved products as indicated by the quality assurance bit flags. In a 32-bit integer, if bit 0, bit 1, bit 2, bit 3, and bit 4 are equal to 1, then these conditional values in profiles are not removed from the analysis (see SAGE-III/ATBD[28]). The limitation of solar radio-occultation measurement inherently influences the number of observations for a given season and latitude and altitude, primarily due to quality assurance during the retrieval of the products. To assess the influence of this issue on the number of observations in each season, we analyzed the seasonal- and zonal-mean latitude distribution of number of counts associated with stratospheric water vapor, ozone and 521 nm aerosol extinction coefficient. We applied nearest-neighbor interpolation (KD tree technique[37]) in each season to partially removed the sampling biases. These sampling biases can arise from various factors, such as viewing geometry of SAGE-III/ISS, causing an uneven spatial/temporal coverage of measurements and data gaps due to satellite operational issues onboard ISS.

We used MLS (Supplementary Figures 11-14) and SAGE III (Supplementary Figure 3, 9) observations to confirm the perturbations in H_2O and O_3 caused by the Hunga eruption. In Supplementary Figure 2 and Supplementary Table 1, we compared the 2022 SAOD values retrieved from SAGE III calculations between lower and upper stratosphere with those from OMPS-NASA, OMPS-SASK, SAGE-III (v5.3, team), OSIRIS, and GloSSAC. Note that OMPS-NASA, OMPS-SASK, SAGE-III (v5.3, team), OSIRIS, and GloSSAC datasets are obtained from Kovilakam[38] (see their Fig. 9a).

Cloud screening within stratosphere altitude

For the stratospheric constituents within stratosphere, we did not apply cloud screening tests, as cloud occurrence in this altitude range is almost negligible[39]. There is a possibility of ice-rich umbrella clouds within 12 hours from the 15 Jan 2022 Hunga eruption[39], which could influence the aerosol layer. But these clouds dissipated within 10-20 minutes in the stratosphere[39]

(see Supplementary Movie 2 in Gupta[39]), and therefore, we assumed cloud-free aerosol conditions within stratosphere.

Aura-MLS observations

We used the MLS[40] (Microwave Limb Sounder) data to estimate the changes in ozone and other related stratospheric trace species before and after the Hunga eruption between January 2005 and December 2023. We also compared the annual variation in O_3 concentration with the MLS observations, which has spatial coverage between 82°S–82°N and vertical pressure level coverage 100 hPa to 0.1 hPa.

ERA5 (ECMWF Reanalysis) data set

The atmospheric profiles such as temperature, pressure, density from SAGE-III/ISS observations are available from around 8 to 100 km. Therefore, for the radiative transfer calculations, we employed collocated measurement of these atmospheric profiles below 9 km ERA5[41] (ECMWF Reanalysis) datasets.

Sulfate aerosols retrievals

We evaluated the properties of stratospheric sulfate aerosols using a Mie lookup table (LUT[42,43]) for sulfate aerosols with a unimodal log-normal distribution with geometric standard deviation (σ) of 1.2. Specifically, the LUT was designed for stratospheric sulfate aerosols and composed of 75% H_2SO_4 (sulfuric acid) and 25% H_2O (water). This LUT provides the extinction, asymmetry parameter, and single scattering albedo as a function of the effective radius (between 0.2 to 1.3 µm; area-weighted mean radius) and the wavelength (between 0.2/0.550 to 100/0.550 µm). This LUT was regenerated with respect to extinction at 521 nm.

The particle size distribution for unimodal log-normal distribution is expressed as:

$$F(r;\bar{r},\sigma,N_0) = \frac{N_o}{(\sqrt{2\pi})rln\sigma} exp\left(\frac{-ln\,(r/\bar{r})^2}{2\sigma^2}\right) \tag{5}$$

where N_o is the number density (m^{-3}), r is particle radius, $\bar{r}$ is median radius (r). The effective radius is related to $\bar{r}$ as

$$r_{eff} = \bar{r} * exp(5/2 * log^2 \sigma) \tag{6}$$

The extinction at $521nm$ wavelength is:

$$K_{ext}(521nm) = \int Q_{ext}(r, n, k, 521nm)\pi r^2 F(r; \bar{r}, \sigma, N_0)dr \tag{7}$$

Based on the real part (n = 1.43) and imaginary part (k = 1.0000E-08) of the complex refractive index of sulfate aerosol at 521 nm[44], we determined the extinction efficiency factor (Q_{ext} at 521 nm) using Mie theory. We used the SAGE-III based observed extinction coefficient and Mie-table to find the number size distribution as a function of latitude, longitude, and altitude (see Supplementary Figure 7).

Calculations of changes in the radiative heating rates and radiative fluxes: Hunga vs. CLIM periods

The composition and properties of atmospheric gases and aerosol conditions are used to analyze the difference between the upward and downward radiative fluxes at each altitude level. For each grid box, we used this difference in upward and downward fluxes at each altitude level to calculate the radiative heating rate due to each species as:

$$H = \frac{-1}{\rho C_p} \frac{\Delta F_{net}(z)}{\Delta z} \tag{8}$$

where H indicates the heating rate [K s^{-1}], t is time (s), g is the acceleration due to gravity (m s^{-2}), Cp is the specific heat content of air at constant pressure (J kg^{-1} K^{-1}), and F is radiative flux (W m^{-2}). ρ indicates air density. ΔF indicates the radiative flux divergence, that is, the difference between the upward and downward radiative fluxes at each altitude level or pressure level, and Δz is the layer thickness. Here, F_{net} can be expressed as:

$$F_{net}(z,\theta,\phi,t) = F^{\downarrow}(z,\theta,\phi,t) - F^{\uparrow}(z,\theta,\phi,t) \tag{9}$$

where $F^{\downarrow}(z,\theta,\phi,t)$ and $F^{\uparrow}(z,\theta,\phi,t)$ are:

$$F^{\uparrow}(\tau,\mu,\phi) = F_O^{\uparrow}(\tau,\mu_0)e^{\left(\frac{-\tau}{\mu_0}\right)} + \int_0^{2\pi} d\phi \int_{\tau_0}^{1} \mu I^{dif}\,(\tau,\mu,\phi)d\mu \tag{10}$$

$$F^{\downarrow}(\tau,\mu,\phi) = F_O^{\downarrow}(\tau,\mu_0)e^{\left(\frac{-\tau}{\mu_0}\right)} + \int_0^{2\pi} d\phi \int_{\tau_0}^{1} \mu I^{dif}\,(\tau,-\mu,\phi)d\mu \tag{11}$$

where I^{dif} (diffuse radiance or source function) in Eq. (11) for 1-D plane-parallel and single scattering problem is described in LibRadtran[30-31] as:

$$\begin{aligned} \mu\frac{I^{dif}(\tau,\mu,\phi)}{d\tau} &= I^{dif}(\tau,\mu,\phi) \\ &- \frac{\omega(r)}{4\pi}\int_0^{2\pi} d\phi' \int_{-1}^{1} d\mu' P(\tau,\mu,\phi;\mu',\phi')\, I^{dif}(\tau,\mu',\phi) \\ &- \big(1-\omega(\tau)\big)B[T(\tau)] - \frac{\omega(r)I^0}{4\pi}P(\tau,\mu,\phi;\mu_0,\phi_0)e^{\left(\frac{-\tau}{\mu_0}\right)} \end{aligned} \tag{12}$$

where $I^0(\pi F_0)$ is the solar irradiance at the top-of-the-atmosphere, $d\tau = \beta_{ext}dz$ is the differential optical depth due to extinction ($\beta_{ext} = \beta_{sca} + \beta_{|abs|}$) of radiation by scattering plus absorption in a given layer thickness (dz), $B[T(\tau)]$ is the Planck function, ω is the single scattering albedo, and $P(\tau,\mu,\phi;\mu',\phi')$ is the phase function for light incident at μ'(cosine of zenith angle), ϕ'(azimuth angle) that is scattered at μ,ϕ, which is expressed in LibRadtran model as:

$$P(\tau,\mu,\phi;\mu',\phi') = \sum_{l=0}^{2M-1}(2l+1)g_l(\tau,\lambda), P_l\,(cos\Phi) \tag{13}$$

where Φ is the phase angle; l is the moment from the Legendre polynomial; and M is the total moments.

The asymmetry factor is written as:

$$g_l(\tau,\lambda) = \frac{1}{2}\int_{-1}^{1} P_l(cos\Phi)P(\tau,\Phi,\lambda)d(cos\Phi) \quad (14)$$

where $P(\tau,\Phi)$ is expressed using standard Legendre polynomials. In this study, $\omega(\lambda,\tau)$ and $g(\lambda,\tau)$ in Eqs. (12) and (14) are retrieved for sulfate aerosol particles within stratosphere before and after the eruption using SAGE-III/ISS and Mie LUT.

The simulated 3D seasonal distribution of net radiative effects (NRE) due to changes in the vertical profiles of stratospheric water vapor, ozone and sulfate aerosol as a function of latitude (θ) and longitude (ϕ) and seasons (S; DJF, MAM, JJA, SON) between Hunga and CLIM periods are calculated as:

where F_{Net} is the sum of the shortwave and longwave components.

$$NRE_{H_2O}(\theta,\phi,z,S) = F_{Net}(\theta,\phi,z,S)_{H_2O}[Hunga] - F_{Net}(\theta,\phi,z,S)_{H_2O}[CLIM] \quad (15)$$

$$NRE_{O_3}(\theta,\phi,z,S) = F_{Net}(\theta,\phi,z,S)_{O_3}[Hunga] - F_{Net}(\theta,\phi,z,S)_{O_3}[CLIM] \quad (16)$$

$$NRE_{Aero}(\theta,\phi,z,S) = F_{Net}(\theta,\phi,z,S)_{Aero}[Hunga] - F_{Net}(\theta,\phi,z,S)_{Aero}[CLIM] \quad (17)$$

Eqs. (15)-(17) are used to compute the annual- and zonal (latitude versus altitude)-mean and vertical-mean (longitude-latitude) radiative effects of Hunga eruption for three different stratospheric species at TOA.

$$NRE_{Combined} = NRE_{H_2O} + NRE_{O_{3(2DF)}} + NRE_{Strat.Aero} \quad (18)$$

Finally, using Eq. (18), a combined net radiative effect at TOA and at 16 km the three stratospheric species is determined by summing up the corresponding net radiative response of each stratospheric species associated with the Hunga eruption. Note that the background atmospheric profiles (such as temperature, density, and pressure only) for both Hunga and CLIM periods are fixed to bring out the instantaneous radiative response of H_2O, $O_{3(2DF)}$ and stratospheric aerosols under idealized conditions, which form one of the limitations of this study.

Supplementary Figures

Supplementary Figure 1

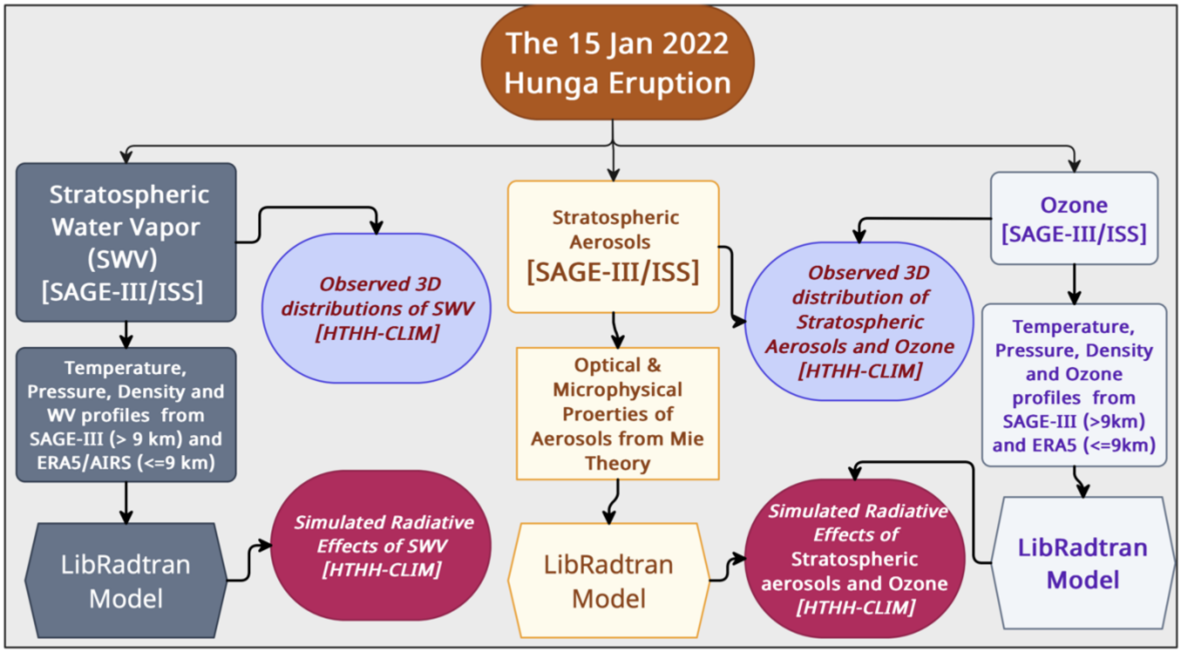

Supplementary Figure 1. Schematic summarizing the methods used to determine the net radiative effects of three stratospheric species—SAOD, SH_2O, and ozone—associated with the 15 January 2022 Hunga eruption. The left-side blocks pertain to SWV, the middle blocks correspond to stratospheric aerosols, and the right-side blocks relate to stratospheric ozone.

Supplementary Figure 2

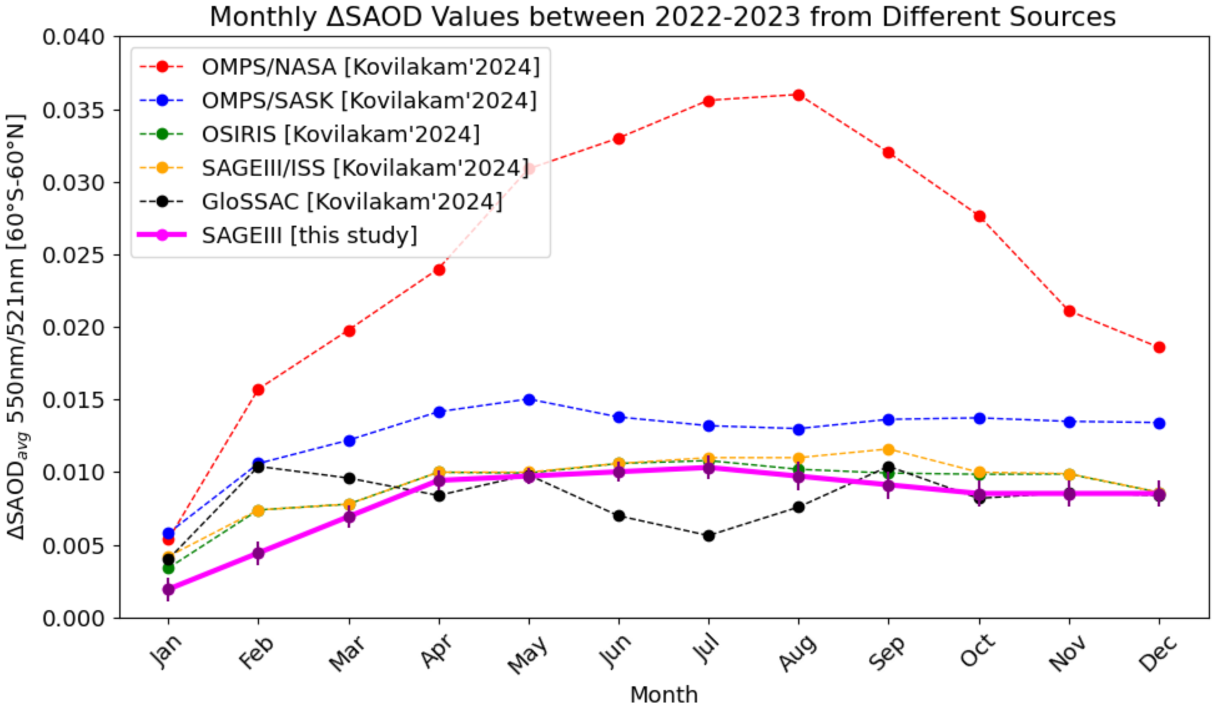

Supplementary Figure 2: Time series of monthly-averaged (60°S–60°N) absolute changes in the 550 nm stratospheric aerosol optical depth (ΔSAOD) during 2022 following the Hunga Tonga–Hunga Ha'apai eruption, relative to background values. The near-global mean ΔSAOD values are obtained from multiple datasets presented in Kovilakam et al. (2024, their Figure 9a): OMPS-LP (NASA) (dashed red line), derived from the Ozone Mapping and Profiler Suite Limb Profiler using a NASA-developed retrieval algorithm; OMPS-LP (University of Saskatchewan) (solid blue line), based on a retrieval algorithm developed by the University of Saskatchewan; OSIRIS (green dashed line), from the Optical Spectrograph and Infrared Imaging System; SAGE-III/ISS (yellow line), from the Stratospheric Aerosol and Gas Experiment III aboard the International Space Station; and GloSSAC (black dashed line), from the Global Space-based Stratospheric Aerosol Climatology dataset. The purple line represents the monthly-averaged (60°S–60°N) absolute changes in ΔSAOD estimated within the stratosphere from this study.

Supplementary Figure 3

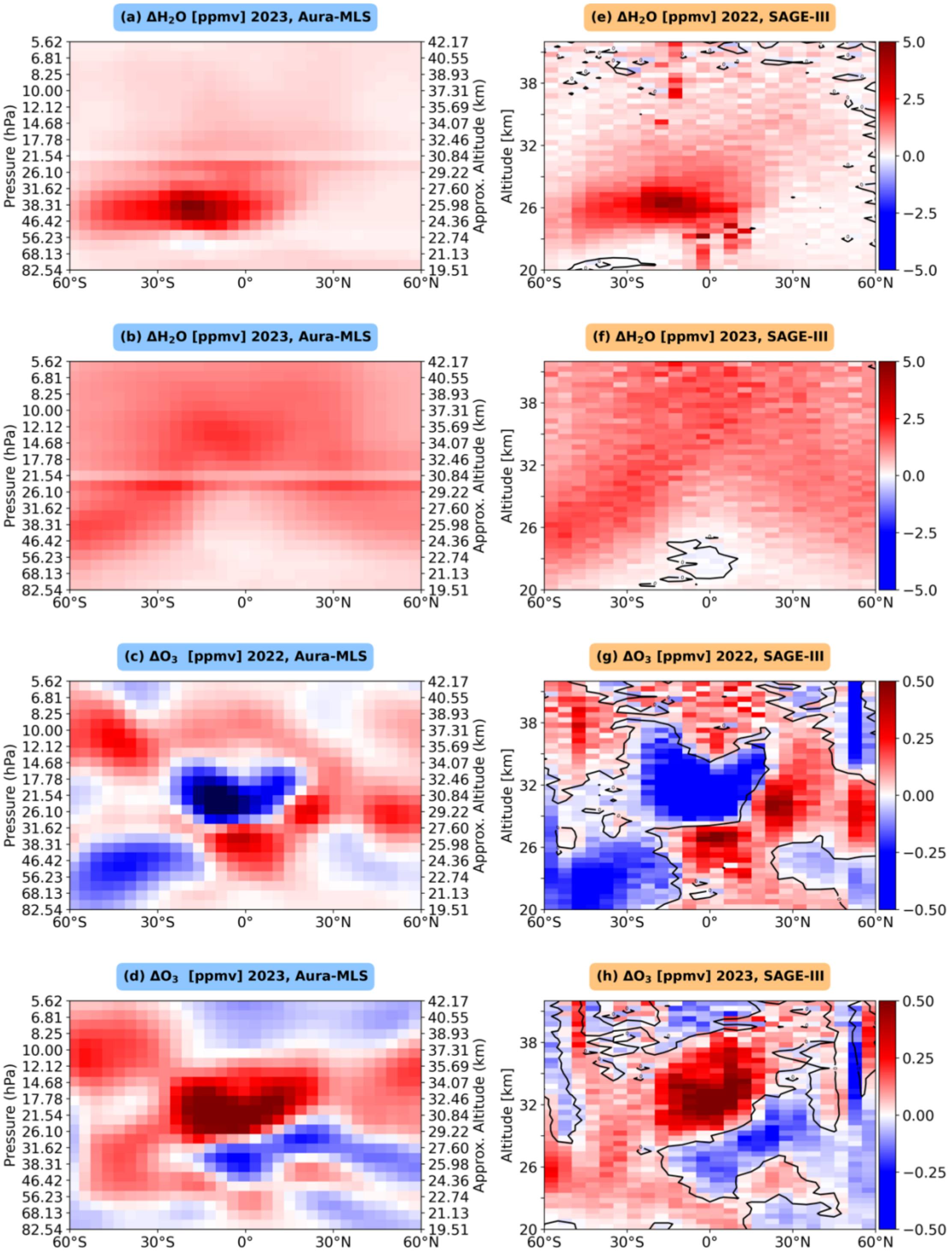

Supplementary Figure 3: Seasonal mean absolute changes in the zonal mean water vapor (SWV) mixing ratio and the corresponding unfiltered ozone mixing ratio for 2022 (first year) versus 2023 (second year), based on Aura-MLS and SAGE-III data. The SWV mixing ratio changes are computed relative to background values (2005–2021) using MLS datasets

(panels **a, b**) and relative to background values (2017–2021) using SAGE-III datasets (panels **c, d**). The ozone mixing ratio changes are determined in the same manner for MLS (panels **a, b**) and SAGE-III (panels **c, d**).

Supplementary Figure 4

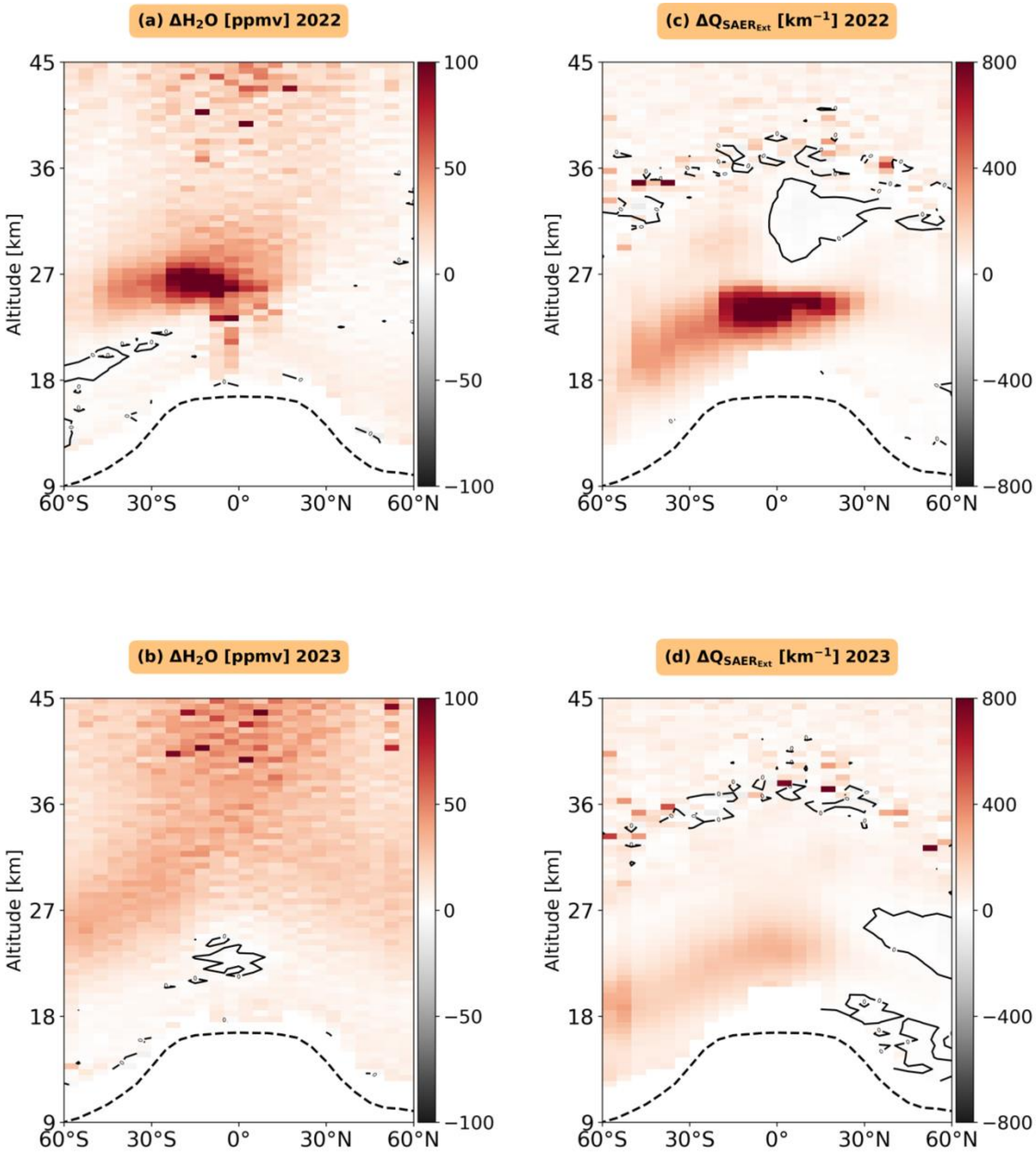

Supplementary Figure 4: Comparison of observed relative changes in three radiatively important stratospheric species between 2022 and 2023 following the Hunga eruption. Panel (**a**) shows the zonal- and annual-mean latitude–altitude variation of the relative change in

the stratospheric water vapor (H_2O) mixing ratio [ppmv] for 2022 relative to the reference (CLIM) period from 07 June 2017 to 09 Dec 2021, while panel (**b**) shows the corresponding changes for 2023 (14 Jan 2022 to 31 Dec 2023). Panel (**c**) shows the stratospheric aerosol extinction coefficient ($Q_{SAERext}$; km^{-1}) at 521 nm from SAGE-III/ISS in 2022, and panel (**d**) presents the data for 2023.

Supplementary Figure 5

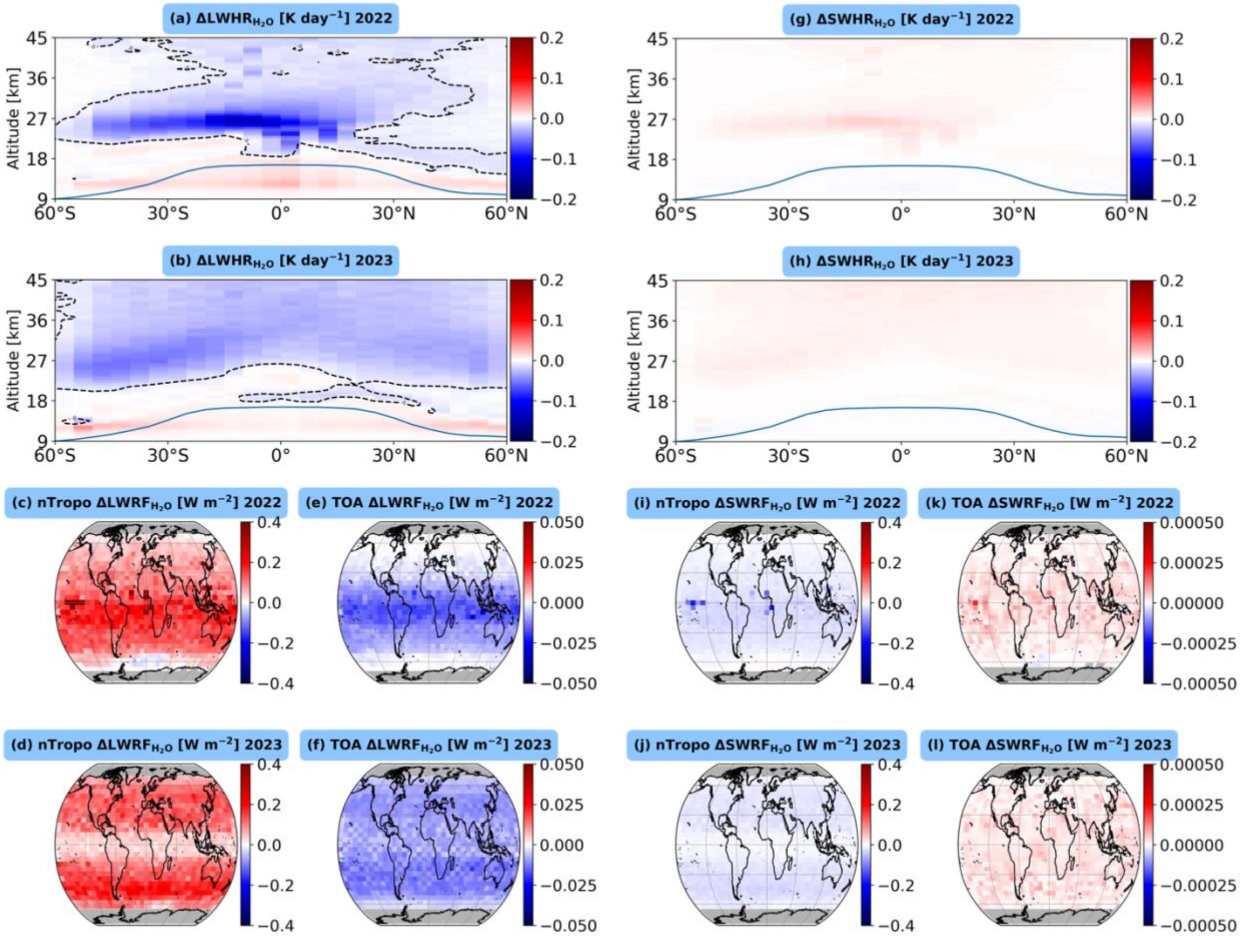

Supplementary Figure 5: Simulated annual-mean stratospheric water vapor radiative heating rates and radiative forcing near the tropopause and at the TOA following the Hunga eruption. Panels (a) and (b) show the latitude–altitude distribution of the differences in longwave radiative heating rates (ΔLWHR; K day^{-1}) within the stratosphere for 2022 and 2023, respectively, relative to the reference (CLIM) period. Panels (c) and (d) depict the changes in

near-tropopause net longwave radiative flux ($\Delta LWRF_{H_2O}$; W m^{-2}) for the same years, highlighting perturbations primarily observed near the lowest levels of the lower stratosphere and just above the tropopause. Panels (c) and (d) display the changes in the TOA longwave radiative flux ($\Delta LWRF_{H_2O}$; W m^{-2}) for 2022 and 2023, respectively. Panels (g) and (h) show the latitude–altitude distribution of the differences in shortwave radiative heating rates (ΔSWHR; K day^{-1}) within the stratosphere for 2022 and 2023, respectively, relative to the CLIM period. Finally, panels (k) and (l) depict the corresponding changes in near-tropopause net shortwave radiative flux ($\Delta SWRF_{H_2O}$; W m^{-2}) for 2022 and 2023, and panels (i) and (j) display the changes in the TOA shortwave radiative flux ($\Delta SWRF_{H_2O}$; W m^{-2}).

Supplementary Figure 6

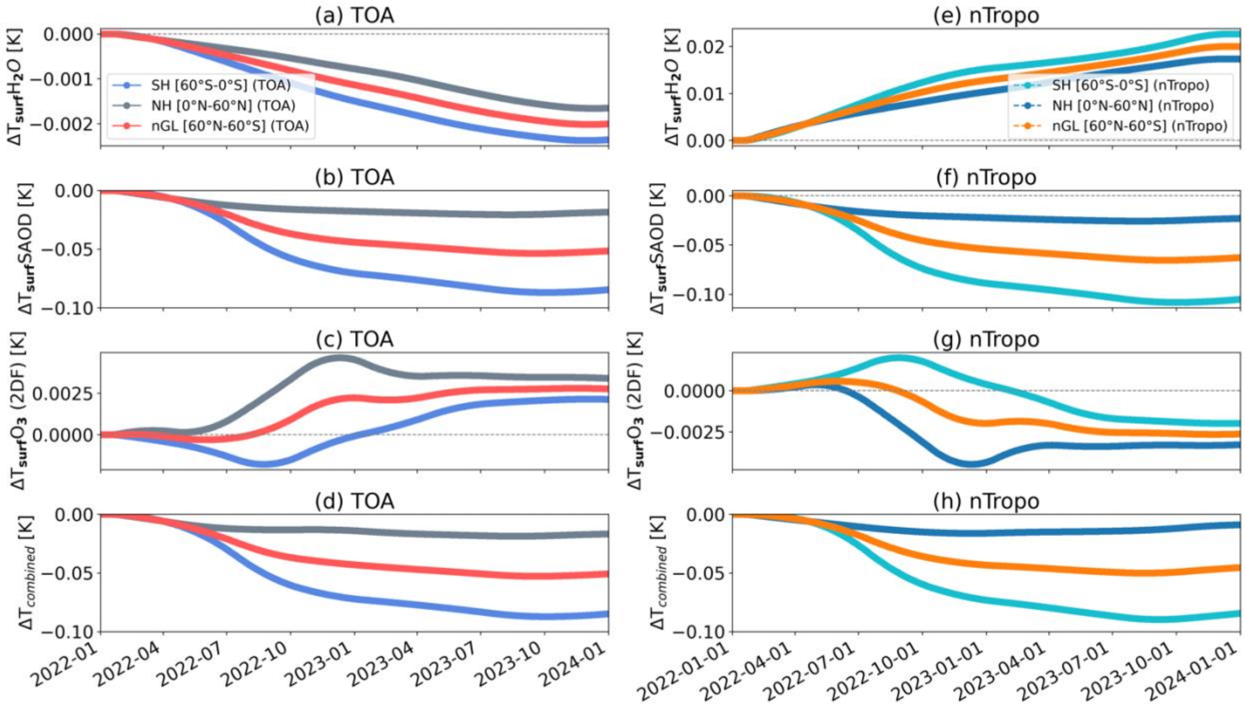

Supplementary Figure 6: Seasonal time series of surface temperature anomalies. Seasonal time series of surface temperature anomalies for a 2-year period (2022 and 2023) following the Hunga eruption, as estimated from instantaneous clear-sky TOA and near the tropopause radiative forcing inputs to the FaIR model for (a, e) SWV only, (b, f) SAOD only, (c, g) ozone (2D-filtered) only, and (d, h) combined values. Note that these surface temperature anomalies are

derived from instantaneous clear-sky radiative forcing values obtained in an idealized simulation and thus represent only a first-order estimate of the actual surface temperature changes.

Supplementary Figure 7

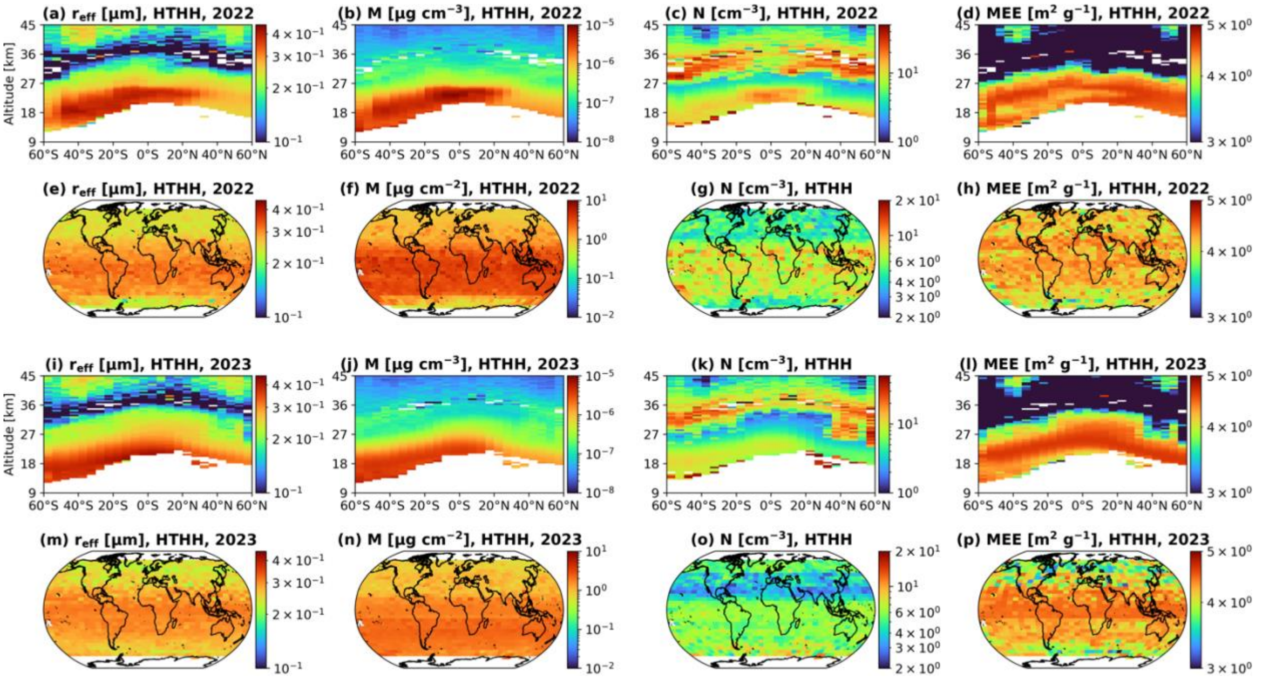

Supplementary Figure 7: Retrieved optical and microphysical properties of sulfate aerosols based on Mie theory. Panels (a)–(d) show the zonal-mean latitude–altitude distribution for the first year (2022) of stratospheric sulfate aerosols: (a) mean effective radius (r_{eff}; μm), (b) mean effective aerosol mass of H_2SO_4 (M_{eff}; μg cm^{-3}), (c) effective aerosol number density (N_{eff}; cm^{-3}), and (d) mass extinction efficiency (MEE; m^2 g^{-1}). Panel (e) presents the mean near-global map of reff (μm), panel (f) displays the vertically integrated effective aerosol mass (M_{eff}; μg cm^{-2}), and panel (g) shows the mean near-global map of N_{eff} (cm^{-3}). Similarly, panels (i)–(l) and (m)–(p) present the corresponding results for the second year (2023).

Supplementary Figure 8

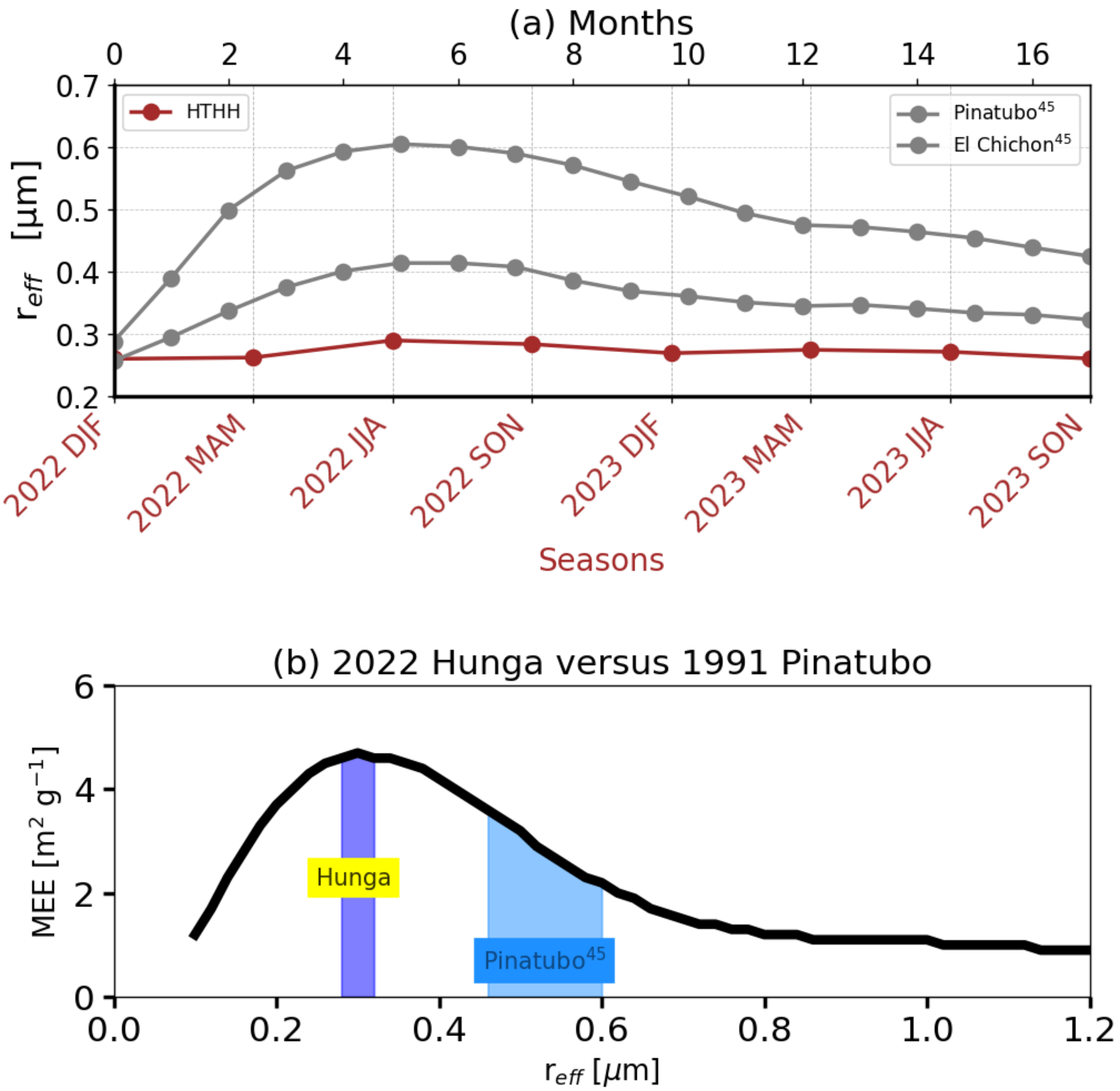

Supplementary Figure 8: Seasonal evolution of the effective radius of sulfate aerosol particles in the tropical region (25°S–25°N) following the Hunga eruption. (a) The brown line with solid dots shows the evolution from 2022 to 2023. For comparison, data on the effective radius of sulfate aerosol particles from the Pinatubo and El Chichón eruptions (Pitari[45]) are also included. (b) The mass extinction efficiency at 521 nm is plotted as a function of effective radius.

Supplementary Figure 9

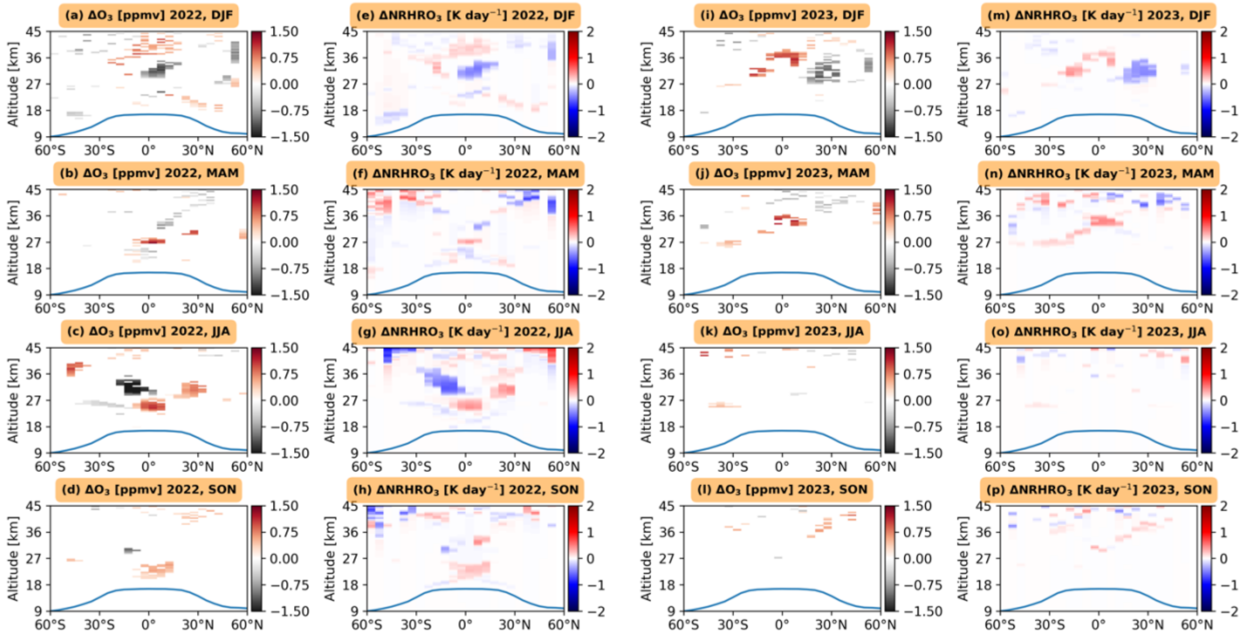

Supplementary Figure 9: Seasonal variations in the observed 2D-filtered changes in stratospheric ozone mixing ratios and the associated simulated instantaneous radiative effects during 2022 and 2023 following the Hunga eruption. Panels (a–d) show the zonal- and seasonal-mean latitude–altitude variation of the changes in stratospheric ozone mixing ratios for 2022, while panels (e–h) show the corresponding latitude–altitude distribution of the differences in net (longwave + shortwave) radiative heating rates (ΔNRHR) for stratospheric ozone in 2022. Panels (i–l) are analogous to (a–d) for 2023, and panels (m–p) correspond to (e–h) for 2023. The method for detecting the clear ozone-perturbed signal is described in the Methods section.

Supplementary Figure 10

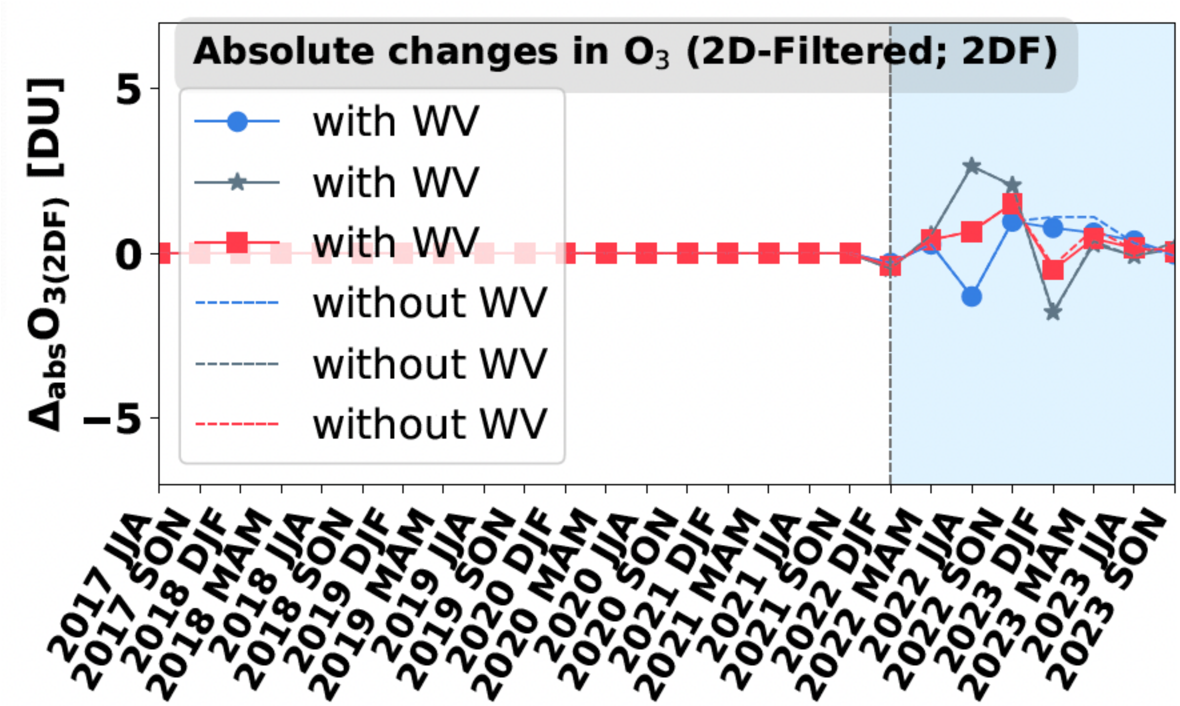

Supplementary Figure 10: Seasonal changes in 2D-filtered ozone between 2017 and 2023. We show the seasonal evolution of 2D-filtered ozone before and after the eruption for the Southern Hemisphere (SH), Northern Hemisphere (NH), and near-global (nGL) regions under conditions with (solid lines) and without (dashed lines) stratospheric water vapor perturbations.

Supplementary Figure 11

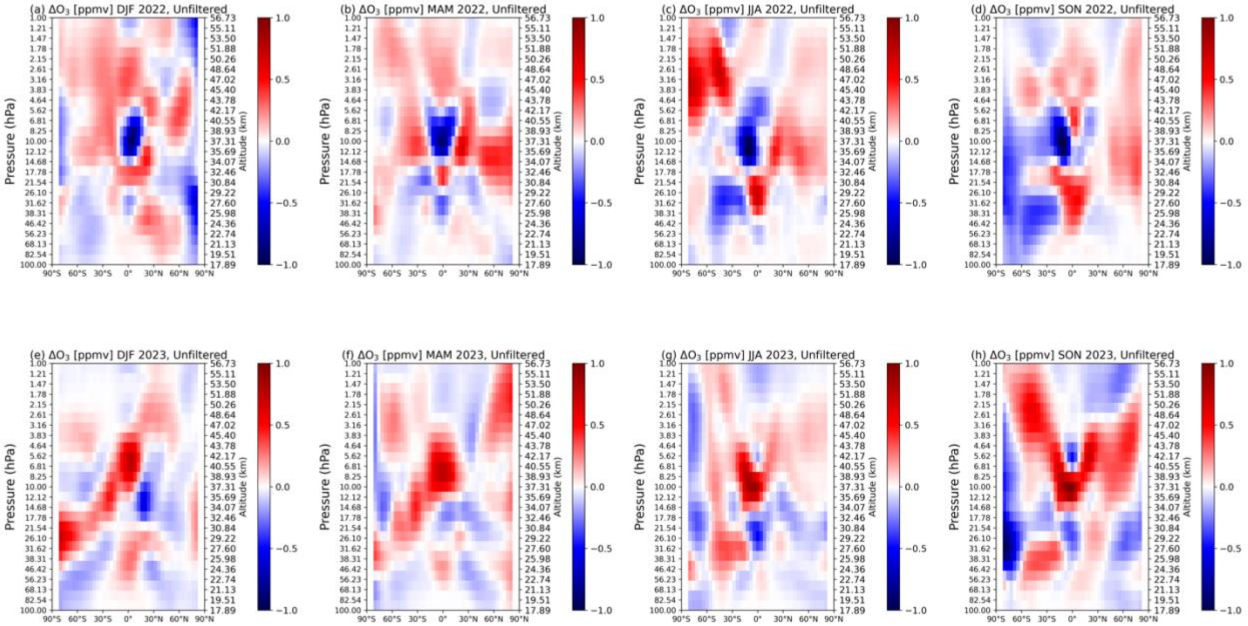

Supplementary Figure 11: Aura-MLS-based seasonal changes in the unfiltered ozone mixing ratio. Aura-MLS observed zonal and seasonal-mean ozone mixing ratio anomalies during summer, winter, spring, and fall for (a–d) 2022 and (e–f) 2023 following the Hunga eruption, relative to the 17-year background mean (2005–2021).

Supplementary Figure 12

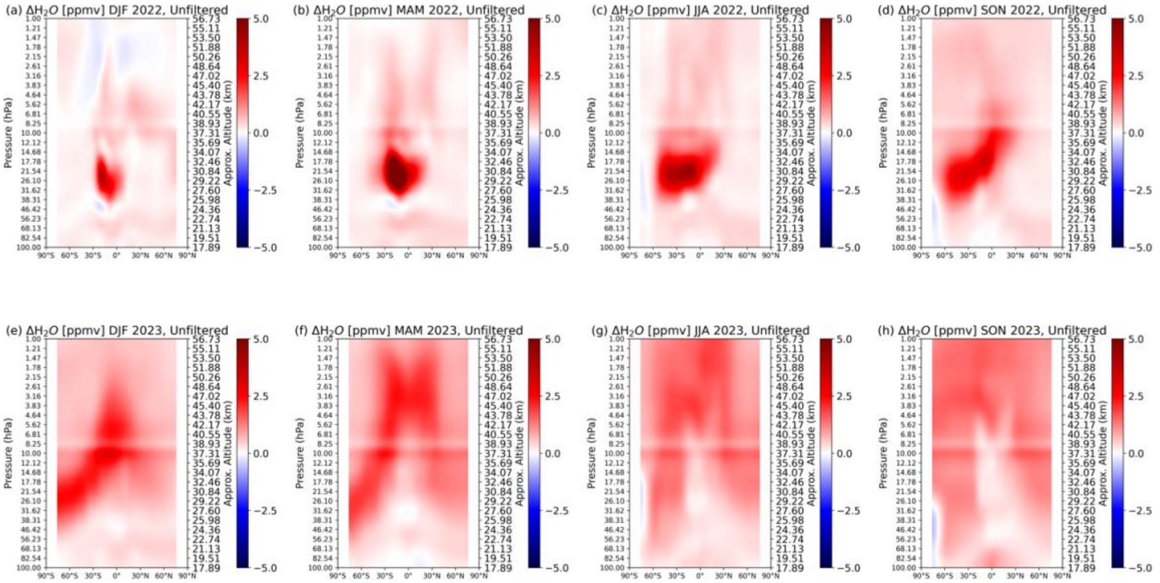

Supplementary Figure 12: Aura-MLS-based seasonal changes in the unfiltered water vapor mixing ratio. Aura-MLS observed zonal and seasonal-mean water vapor mixing ratio anomalies during summer, winter, spring, and fall for (a–d) 2022 and (e–f) 2023 following the Hunga eruption, relative to the 17-year background mean (2005–2021).

Supplementary Figure 13

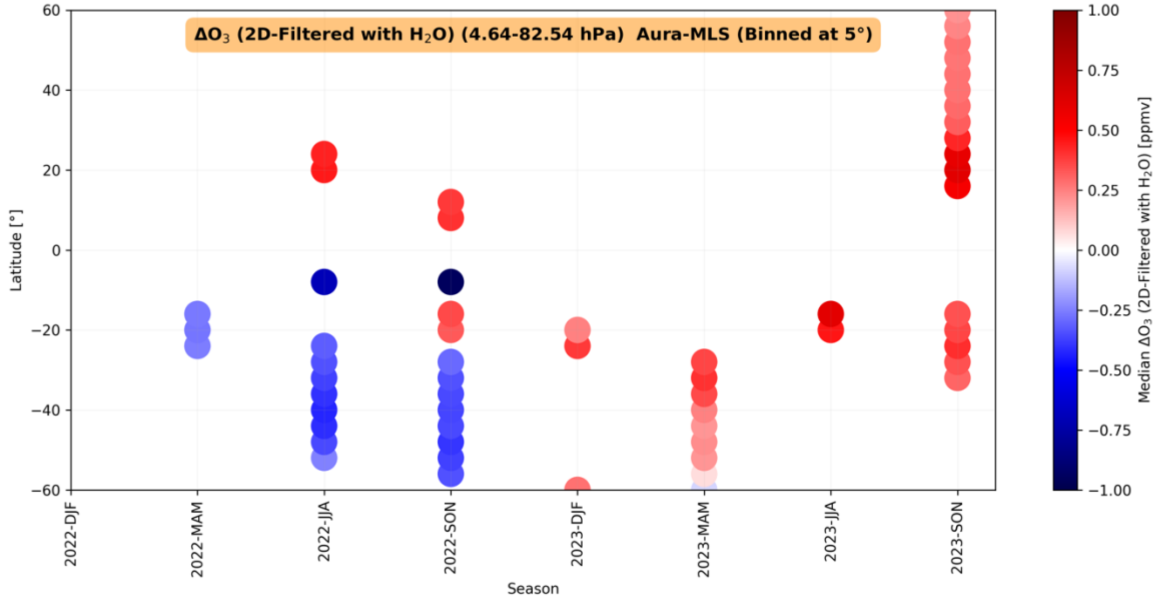

Supplementary Figure 13: Aura-MLS observed zonal-mean filtered stratospheric ozone anomalies. Aura-MLS observed zonal-mean, 2D-filtered (with water vapor at or above 2σ)

seasonal time series of median changes in stratospheric ozone mixing ratio anomalies between 4.64 and 82.54 hPa as a function of season and latitude (binned at 5°) during 2022 and 2023 following the Hunga eruption. The anomalies are calculated relative to background mean values from 2005 to 2021 obtained from the MLS datasets.

Supplementary Figure 14

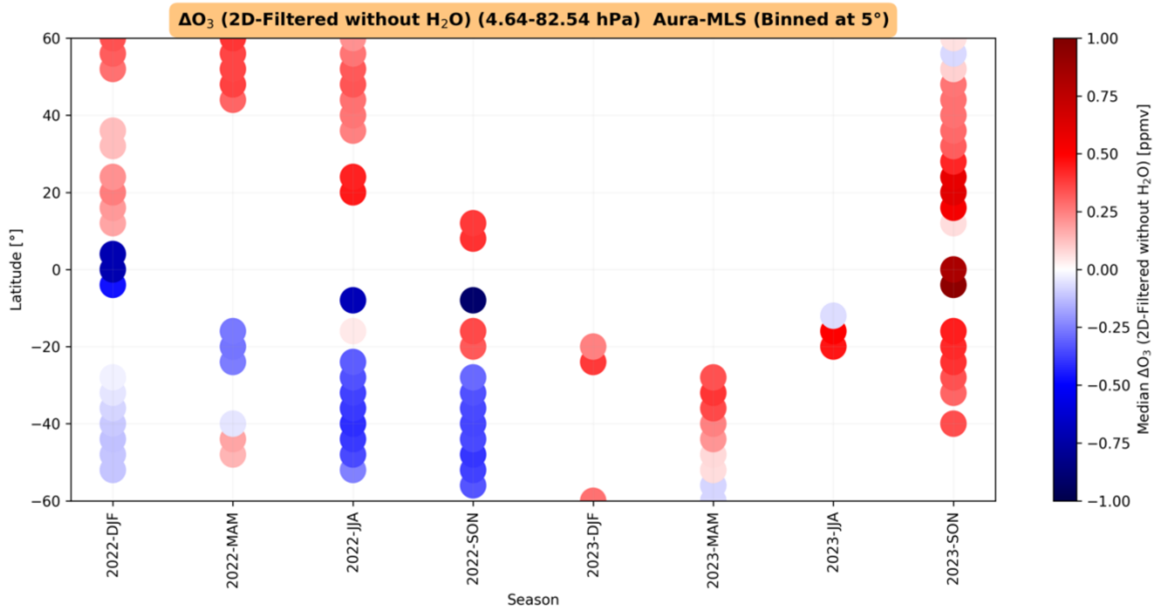

Supplementary Figure 14: Aura-MLS observed zonal-mean 2D-filtered stratospheric ozone anomalies (with no water vapor filter). Aura-MLS observed zonal-mean, 2D-filtered (without the water vapor condition) seasonal time series of median changes in stratospheric ozone mixing ratio anomalies between 4.64 and 82.54 hPa as a function of season and latitude (binned at 5°) during 2022 and 2023 following the Hunga eruption. The anomalies are calculated relative to background mean values from 2005 to 2021 obtained from the MLS datasets.

Supplementary Figure 15

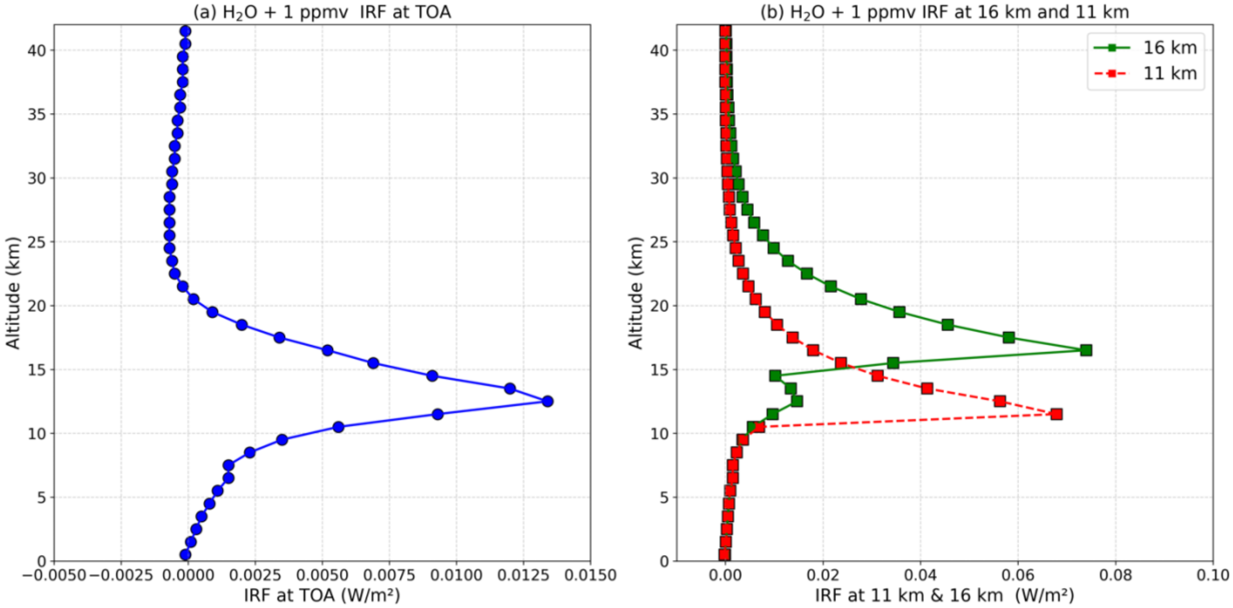

Supplementary Figure 15: LW instantaneous radiative forcing kernels of water vapor. (**a**) LW instantaneous radiative forcing kernel of water vapor with a +1 ppmv perturbation per km at the TOA (blue line). (**b**) LW instantaneous radiative forcing kernels of water vapor with a +1 ppmv perturbation per km at 16 km (green) and 11 km (red). Calculations are performed for the midlatitude summer standard atmosphere.

Supplementary Figure 16

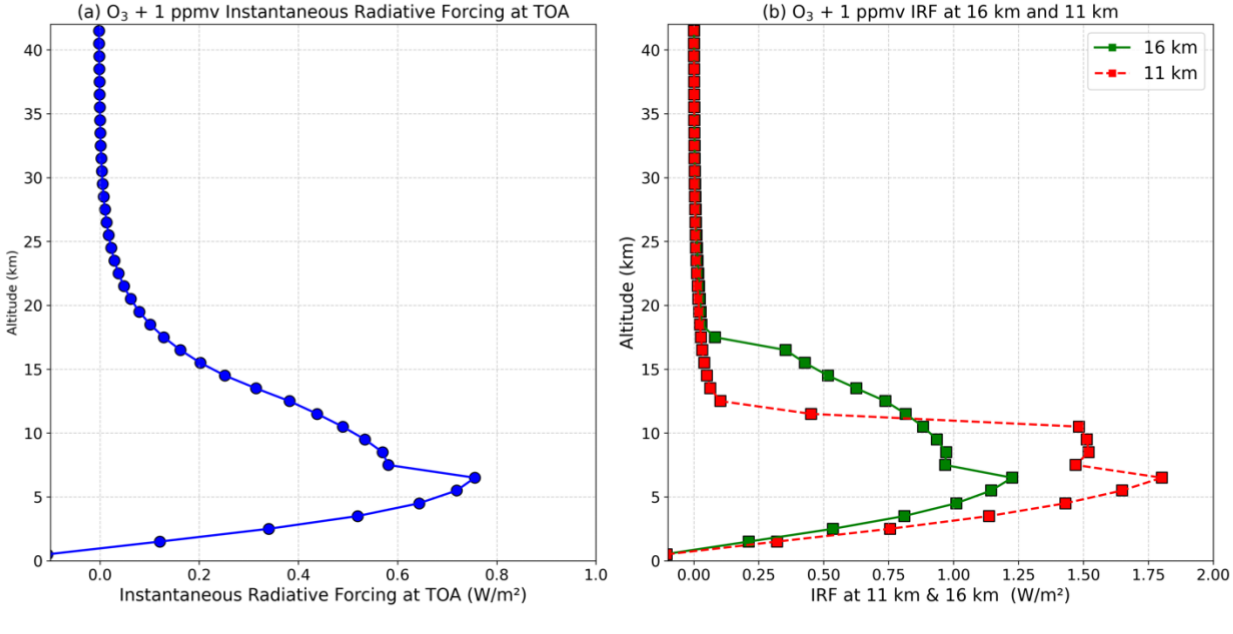

Supplementary Figure 16: LW instantaneous radiative forcing kernels of ozone. (a) LW

instantaneous radiative forcing kernel of ozone with a +1 ppmv perturbation per km at the TOA (blue line) and (b) at 16 km (green) and 11 km (red). Calculations are performed for the midlatitude summer standard atmosphere. Note that SW instantaneous radiative forcing dominates over LW instantaneous radiative forcing for ozone.

Supplementary Figure 17

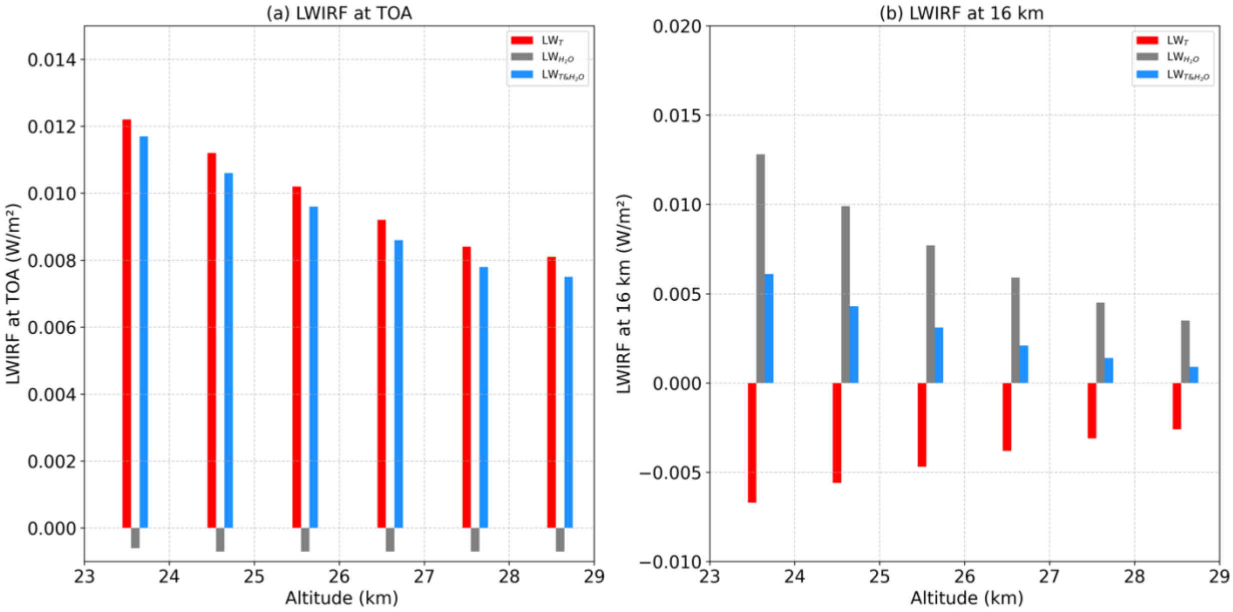

Supplementary Figure 17: LW instantaneous radiative forcing of perturbed temperature, water vapor, and combined Effects. (a) LW instantaneous radiative forcing (IRF) at the TOA as a function of altitude (23–29 km): Grey bars: IRF of water vapor with a +1 ppmv per km perturbation. Red bars: IRF of temperature change at 1 K per km. Blue bars: IRF for the combined perturbation (water vapor: +1 ppmv per km; temperature: 1 K per km). (b) The corresponding IRF at 16 km, with grey, red, and blue bars representing water vapor, temperature, and combined perturbations, respectively.

Supplementary Figure 18

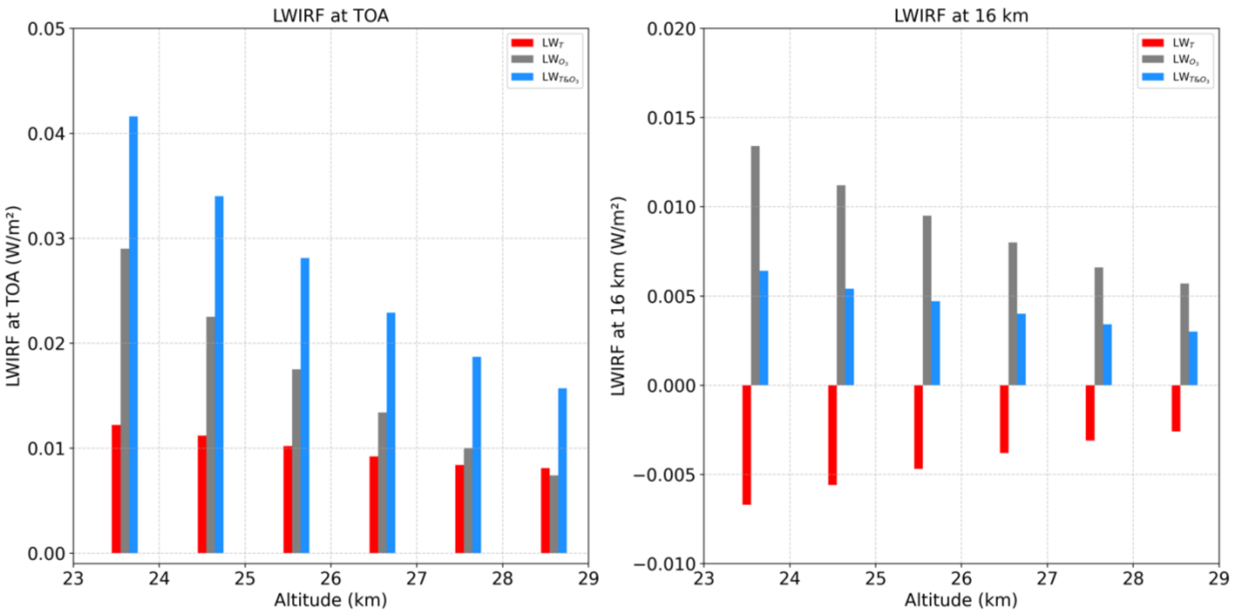

Supplementary Figure 18: LW instantaneous radiative forcing of perturbed temperature, ozone, and combined effects. (a) LW instantaneous radiative forcing (IRF) at the TOA for altitudes between 23 and 29 km: Grey bars: IRF of ozone with a +1 ppmv per km perturbation. Red bars: IRF of temperature change at 1 K per km. Blue bars: IRF for the combined perturbation (ozone: +1 ppmv per km; temperature: 1 K per km). (b) The corresponding IRF at 16 km, with grey, red, and blue bars representing ozone, temperature, and combined perturbations, respectively.

Supplementary Figure 19

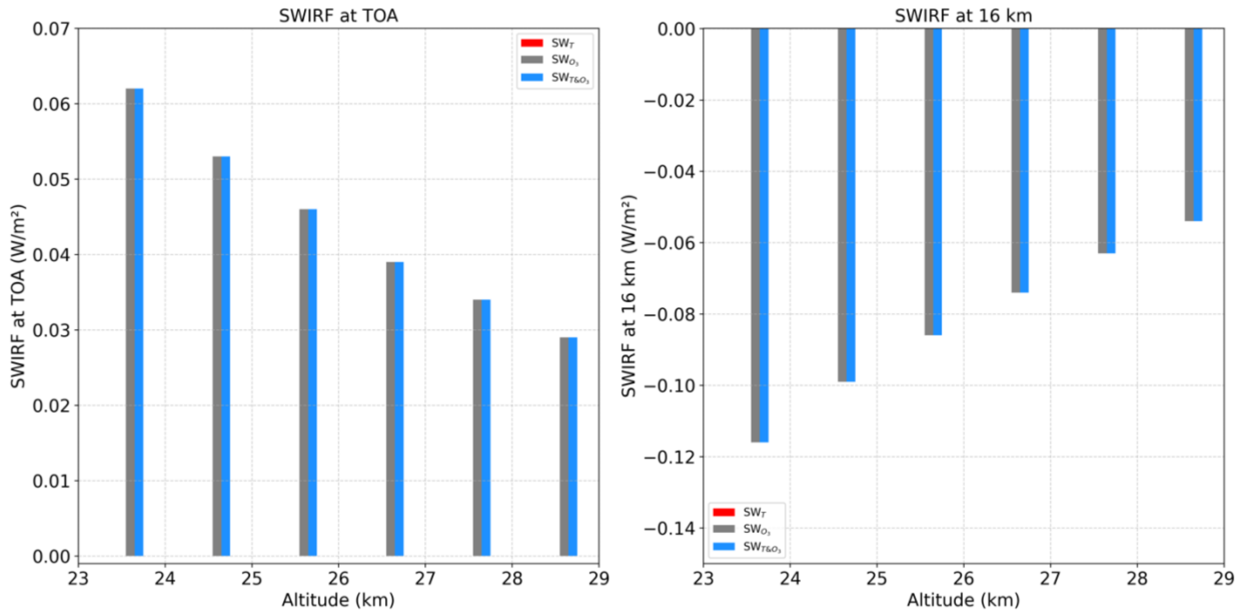

Supplementary Figure 19: SW Instantaneous Radiative Forcing of Perturbed Temperature, Ozone, and Combined Effects. Panels (a) and (b) present the SW instantaneous radiative forcing (IRF) as a function of altitude (23–29 km) for: Ozone perturbation: +1 ppmv per km (grey bars) at the TOA (panel a) and at 16 km (panel b). Temperature perturbation: 1 K per km (red bars) at the TOA (panel a) and at 16 km (panel b). Combined perturbation: both ozone (+1 ppmv per km) and temperature (1 K per km) (blue bars) at the TOA (panel a) and at 16 km (panel b).

Supplementary Table 1

Different instruments/methods for SAOD values during 2022 after Hunga eruption	The 2022 annual mean percentage change (%) w. r. t. to ΔSAOD within stratosphere from SAGE-III
**OMPS/NASA**	-66.5
**OMPS/SASK**	-37.8
**OSIRIS**	-12.6
**SAGEIII/ISS team**	-15.5
**GloSSAC**	1.8

Supplementary Table 1: Annual mean percentage change in near-global stratospheric aerosol optical depth (ΔSAOD) in 2022. This table presents the annual mean percentage change in the near-global (60°S–60°N) absolute stratospheric aerosol optical depth (ΔSAOD) for each instrument (including data from the SAGE-III/ISS team), relative to SAGE-III/ISS-based data within the stratosphere.

Supplementary References

1. Jenkins, Stuart, Chris Smith, Myles Allen, and Roy Grainger. Tonga eruption increases chance of temporary surface temperature anomaly above 1.5° C. Nature Climate Change13, 2, 127-129 (2023).

2. Sellitto, P. et. al. The Unexpected Radiative Impact Of The Hunga Tonga Eruption Of 15 January 2022. Communications Earth & Environment 1, 288 (2022).

3. Günther, A., 2018. Stratospheric sulphur: MIPAS/Envisat measurements and chemical transport model simulations of carbonyl sulphide, sulphur dioxide, and sulphate aerosol.

4. Duchamp, C., Wrana, F., Legras, B., Sellitto, P., Belhadji, R. and von Savigny, C., 2023. Observation of the aerosol plume from the 2022 Hunga Tonga—Hunga Ha'apai eruption with SAGE III/ISS. *Geophysical Research Letters*, *50*(18), p.e2023GL105076.

5. Schoeberl, M. The Estimated Climate Impact of the Hunga Tonga-Hunga Ha'apai Eruption Plume. *Geophysical Research Letters* 50, 18: e2023GL104634 (2023).

6. Zhu, Y. et. al. Perturbations in stratospheric aerosol evolution due to the water-rich plume of the 2022 Hunga-Tonga eruption. Communications Earth & Environment, 3(1), 248 (2022).

7. Schoeberl, M., Wang, Y., Taha, G., Zawada, D.J., Ueyama, R. and Dessler, A., 2024. Evolution of the Climate Forcing During the Two Years after the Hunga Tonga-Hunga Ha'apai Eruption. *Authorea Preprints*.

8. Wilmouth, David M., Freja F. Østerstrøm, Jessica B. Smith, James G. Anderson, and Ross J. Salawitch. "Impact of the Hunga Tonga volcanic eruption on stratospheric composition." *Proceedings of the National Academy of Sciences* 120, no. 46 (2023): e2301994120.

9. Santee, M.L. et. al., 2023. Strong Evidence of Heterogeneous Processing on Stratospheric Sulfate Aerosol in the Extrapolar Southern Hemisphere Following the 2022 Hunga Tonga-Hunga Ha'apai Eruption. *Journal of Geophysical Research: Atmospheres*, *128*(16), p.e2023JD039169.

10. Wang, X. et. al. 2023. Stratospheric Climate Anomalies and Ozone Loss Caused by the Hunga Tonga-Hunga Ha'apai Volcanic Eruption. *Journal of Geophysical Research: Atmospheres*, *128*(22), p.e2023JD039480.

11. Zhang, Jun, Douglas Kinnison, Yunqian Zhu, Xinyue Wang, Simone Tilmes, Kimberlee Dube, and William Randel. "Chemistry contribution to stratospheric ozone depletion after the unprecedented water-rich Hunga Tonga eruption." *Geophysical Research Letters* 51, no. 7 (2024): e2023GL105762.

12. Asher, E., Todt, M., Rosenlof, K., Thornberry, T., Gao, R.S., Taha, G., Walter, P., Alvarez, S., Flynn, J., Davis, S.M. and Evan, S., 2023. Unexpectedly rapid aerosol formation in the Hunga Tonga plume. *Proceedings of the National Academy of Sciences*, *120*(46), p.e2219547120

13. Legras, B., Duchamp, C., Sellitto, P., Podglajen, A., Carboni, E., Siddans, R., Grooß, J.U., Khaykin, S. and Ploeger, F., 2022. The evolution and dynamics of the Hunga Tonga–Hunga Ha'apai sulfate aerosol plume in the stratosphere. *Atmospheric Chemistry and Physics*, *22*(22), pp.14957-14970.

14 Smith, C.J., et. al. FAIR v1. 3: a simple emissions-based impulse response and carbon cycle model. Geoscientific Model Development, 11(6), 2273-2297 (2018).

15. Carslaw, K. S. et. al. Stratospheric aerosol growth and HNO3 gas phase depletion from coupled HNO3 and water uptake by liquid particles. Geophys. Res. Lett., 21(23), 2479-2482 (1994).

16. Solomon, S., Garcia, R.R., Rowland, F.S. and Wuebbles, D.J. On the depletion of Antarctic ozone. Nature, 321(6072), 755-758 (1986).

17. Lu, J. et. al. Stratospheric Aerosol and Ozone Responses to the Hunga Tonga-Hunga Ha'apai Volcanic Eruption. Geophys. Res. Lett., 50(4), e2022GL102315 (2023).

18. Evan, Stephanie, et al. Rapid ozone depletion after humidification of the stratosphere by the Hunga Tonga Eruption. *Science* 382, 6668: eadg2551 (2023).

19. National Academies of Sciences, Engineering, and Medicine. Radiative Forcing of Climate Change: Expanding the Concept and Addressing Uncertainties. Washington, DC: The National Academies Press. https://doi.org/10.17226/11175 (2005).

20. Shine, K.P., Byrom, R.E. and Checa-Garcia, R. Separating the shortwave and longwave components of greenhouse gas radiative forcing. Atmos. Science Letters, 23(10), e1116 (2022).

21. Davis, S.M et. al., 2021. Validation of SAGE III/ISS solar water vapor data with correlative satellite and balloon-borne measurements. *Journal of Geophysical Research: Atmospheres*, *126*(2), p.e2020JD033803.

22. Wang, H.R. et. al., 2020. Validation of SAGE III/ISS solar occultation ozone products with correlative satellite and ground-based measurements. *Journal of Geophysical Research: Atmospheres*, *125*(11), p.e2020JD032430.

23. Stenchikov, G. et. al. How does a Pinatubo-size volcanic cloud reach the middle stratosphere? Journal of Geophysical Research: Atmospheres, 126(10), e2020JD033829 (2021).

24. Gerlach, T.M., Westrich, H.R. and Symonds, R.B. Preeruption vapor in magma of the climactic Mount Pinatubo eruption: Source of the giant stratospheric sulfur dioxide cloud. Fire and mud: eruptions and lahars of Mount Pinatubo, Philippines, 415, 33 (1996).

25. LeGrande, A.N., Tsigaridis, K. and Bauer, S.E. Role of atmospheric chemistry in the climate impacts of stratospheric volcanic injections. Nature Geoscience, 9(9), 652-655 (2016).

26. Solomon, S. et. al. Contributions of stratospheric water vapor to decadal changes in the rate of global warming. *Science*, *327*(5970), 1219-1223, (2010).

27. Wang, Y. and Huang, Y. Compensating atmospheric adjustments reduce the volcanic forcing from Hunga stratospheric water vapor enhancement. *Science Advances*, *10*(32), p.eadl2842 (2024).

28. SA, E. SAGE III, https://eospso.gsfc.nasa.gov/sites/default/files/atbd/atbd-sage-cloud.pdf (2002).

29. LibRadtran document, http://www.libradtran.org/doc/libradtran.pdf (see Eq. 2.25), (2005)

30. Mayer, B. & Kylling, A. The libRadtran software package for radiative transfer calculations-description and examples of use. Atmos. Chem. Phys. 5, 7, 1855-1877 (2005).

31. Gasteiger, J. et. al.: Representative wavelengths absorption parameterization applied to satellite channels and spectral bands, J. Quant. Spectrosc. Radiat. Transfer, 148, 99–115, doi:10.1016/j.jqsrt.2014.06.024, 2014.

32. Stamnes, K., Tsay, S.C., Wiscombe, W. and Laszlo, I. DISORT, general-purpose Fortran program for discrete-ordinate-method radiative transfer in scattering and emitting layered media: documentation of

33. Haywood, J.M. & Shine, K.P. The effect of anthropogenic sulfate and soot aerosol on the clear sky planetary radiation budget. *Geophys. Res. Lett*, *22*(5), 603-606 (1995).

34. Baker, D. N. & Kanekal, S.G. Solar cycle changes, geomagnetic variations, and energetic particle properties in the inner magnetosphere. *Journal of Atmospheric and Solar-Terrestrial Physics*, *70*(2-4), 195-206 (2008)

35. Bellouin, N. et. al. Radiative forcing of climate change from the Copernicus reanalysis of atmospheric composition. *Earth System Science Data*, *12*(3),1649-1677 (2020).

36. Tong, W., Li, L., Zhou, X. and Franklin, J. Efficient spatiotemporal interpolation with spark machine learning. Earth Science Informatics, 12, 87-96 (2019).

37. Lauster, B. et. al. Occurrence of polar stratospheric clouds as derived from ground-based zenith DOAS observations using the colour index. Atmospheric Chemistry and Physics, 22(24), 15925-15942 (2022).

38. Kovilakam, Mahesh, Larry Thomason, Magali Verkerk, Thomas Aubry, and Travis Knepp. "OMPS-LP Aerosol Extinction Coefficients And Their Applicability in GloSSAC." *EGUsphere* 2024 (2024): 1-31.

39. Gupta, A.K., Bennartz, R., Fauria, K.E. and Mittal, T. Eruption chronology of the December 2021 to January 2022 Hunga Tonga-Hunga Ha'apai eruption sequence. Communications Earth & Environment, 3(1), 314 (2022).

40. Waters, Joe W. et al. The earth observing system microwave limb sounder (EOS MLS) on the Aura satellite. IEEE transactions on geoscience and remote sensing 44, 5, 1075-1092 (2006).

41. Hersbach, H. et al. The ERA5 global reanalysis. Q. J. R. Meteorol. Soc. **146**, 1999–2049 (2020).

42. Aubry, T.J., Toohey, M., Marshall, L., Schmidt, A. & Jellinek, A.M. A new volcanic stratospheric sulfate aerosol forcing emulator (EVA_H): Comparison with interactive stratospheric aerosol models. J. of Geophys. Res., Atmospheres, 125(3), e2019JD031303 (2020).

43. Toohey, M., Stevens, B., Schmidt, H. and Timmreck, C. Easy Volcanic Aerosol (EVA v1. 0): an idealized forcing generator for climate simulations. Geoscientific Model Development, 9(11), 4049-4070 (2016).

44. Armante, R. et. al. Evaluation of spectroscopic databases through radiative transfer simulations compared to observations. Application to the validation of GEISA 2015 with IASI and TCCON." Journal of Molecular Spectroscopy 327: 180-192 (2016).

45. Pitari, G., et. al. Stratospheric aerosols from major volcanic eruptions: A composition-climate model study of the aerosol cloud dispersal and e-folding time. *Atmosphere*, *7*(6), 75 (2016).
