# Supplementary material for: The January 2022 Hunga eruption cooled the southern hemisphere in 2022 and 2023
Source: Commun Earth Environ. 2025 Mar 27;6(1):240. doi: 10.1038/s43247-025-02181-9 (PMC11949836; doi:10.1038/s43247-025-02181-9)
Supplement: Supplementary file 3 — Description of Additional Supplementary Files [file 43247_2025_2181_MOESM3_ESM.pdf]

Description of Additional Supplementary Files

Data for Figures in the Main Article

- **File Name:** *Supplementary Data 1* (Related to **Figure 1**)
  Description: This dataset corresponds to Figure 1. The processed data can also be accessed using **Gupta**[85].

- **File Name:** *Supplementary Data 2* (Related to **Figure 2**)
  Description: This dataset corresponds to Figure 2. The processed data can also be accessed using Gupta[85].

- **File Name:** *Supplementary Data 3* (Related to **Figure 3**)
  Description: This dataset corresponds to Figure 3. The processed data can also be accessed using Gupta[85].

- **File Name:** *Supplementary Data 4* (Related to **Figure 4**)
  Description: This dataset corresponds to Figure 4. The processed data can also be accessed using Gupta[85].

- **File Name:** *Supplementary Data 5* (Related to **Figure 5**)
  Description: This dataset corresponds to Figure 5. The processed data can also be accessed using Gupta[85].
