# Supplementary material for: The January 2022 Hunga eruption cooled the southern hemisphere in 2022 and 2023
Source: Commun Earth Environ. 2025 Mar 27;6(1):240. doi: 10.1038/s43247-025-02181-9 (PMC11949836; doi:10.1038/s43247-025-02181-9)
Supplement: Supplementary file 5 — reporting summary [file 43247_2025_2181_MOESM5_ESM.pdf]

nature portfolio

Corresponding author(s): COMMSENV-24-1061B

Last updated by author(s): Feb 14, 2025

Reporting Summary

Nature Portfolio wishes to improve the reproducibility of the work that we publish. This form provides structure for consistency and transparency in reporting. For further information on Nature Portfolio policies, see our Editorial Policies and the Editorial Policy Checklist.

Statistics

For all statistical analyses, confirm that the following items are present in the figure legend, table legend, main text, or Methods section.

n/a	Confirmed	
☐	☒	The exact sample size (n) for each experimental group/condition, given as a discrete number and unit of measurement
☒	☐	A statement on whether measurements were taken from distinct samples or whether the same sample was measured repeatedly
☒	☐	The statistical test(s) used AND whether they are one- or two-sided<br>*Only common tests should be described solely by name; describe more complex techniques in the Methods section.*
☐	☒	A description of all covariates tested
☐	☒	A description of any assumptions or corrections, such as tests of normality and adjustment for multiple comparisons
☐	☒	A full description of the statistical parameters including central tendency (e.g. means) or other basic estimates (e.g. regression coefficient) AND variation (e.g. standard deviation) or associated estimates of uncertainty (e.g. confidence intervals)
☒	☐	For null hypothesis testing, the test statistic (e.g. F, t, r) with confidence intervals, effect sizes, degrees of freedom and P value noted<br>*Give P values as exact values whenever suitable.*
☒	☐	For Bayesian analysis, information on the choice of priors and Markov chain Monte Carlo settings
☒	☐	For hierarchical and complex designs, identification of the appropriate level for tests and full reporting of outcomes
☒	☐	Estimates of effect sizes (e.g. Cohen's d, Pearson's r), indicating how they were calculated

Our web collection on statistics for biologists contains articles on many of the points above.

Software and code

Policy information about availability of computer code

Data collection	The SAGE-III/ISS v053 solar datasets (filename: g3bssp_53) are freely available from (https://asdc.larc.nasa.gov/project/SAGE%20III-ISS/g3bssp_53). The Aura-MLS dataset is also freely available and can be obtained using https://search.earthdata.nasa.gov/. The ECMWF ERA5 reanalysis pressure level datasets can be obtained from https://cds.climate.copernicus.eu/cdsapp#!/search?type=dataset. Underlying processed and simulated data set related to the manuscript (Figure 1-4) and supplementary information (Supplementary Figure 1-8) generated using SAGE-III/ISS and MLS and LibRadtran model will be publicly available at zendo website under this account (https://zenodo.org/deposit?page=1&size=20). The data related to the refractive index of sulfate aerosols is taken from https://geisa.aeris-data.fr/litms/. The Mie table for sulfate aerosol is available here: https://github.com/matthew2e/easy-volcanic-aerosol/blob/master/eva_Mie_lookuptables.nc59.
Data analysis	We used The LibRadtran mdoel. LibRadtran model is available at http://www.libradtran.org/doku.php?id=download here. All the Figures (including Supplementary Figures) were originally produced and plotted using various open-source Python libraries (e.g., https://matplotlib.org/stable/). The LibRadtran model-based processed data and input will also be publicly available on the Zenodo website under this account (https://zenodo.org/deposit?page=1&size=20) at the time of publication.

For manuscripts utilizing custom algorithms or software that are central to the research but not yet described in published literature, software must be made available to editors and reviewers. We strongly encourage code deposition in a community repository (e.g. GitHub). See the Nature Portfolio guidelines for submitting code & software for further information.

Data

Policy information about availability of data

All manuscripts must include a data availability statement. This statement should provide the following information, where applicable:

- Accession codes, unique identifiers, or web links for publicly available datasets
- A description of any restrictions on data availability
- For clinical datasets or third party data, please ensure that the statement adheres to our policy

The SAGE-III/ISS v053 solar datasets (filename: g3bssp_53) are freely available from (https://asdc.larc.nasa.gov/project/SAGE%20III-ISS/g3bssp_53). The Aura-MLS dataset is also freely available and can be obtained using https://search.earthdata.nasa.gov/. The ECMWF ERA5 reanalysis pressure level datasets can be obtained from https://cds.climate.copernicus.eu/cdsapp#!/search?type=dataset. Underlying processed and simulated data set related to the manuscript (Figure 1-4) and supplementary information (Supplementary Figure 1-8) generated using SAGE-III/ISS and MLS and LibRadtran model will be publicly available at zendo website under this account (https://zenodo.org/deposit?page=1&size=20). The data related to the refractive index of sulfate aerosols is taken from https://geisa.aeris-data.fr/litms/. The Mie table for sulfate aerosol is available here: https://github.com/matthew2e/easy-volcanic-aerosol/blob/master/eva_Mie_lookuptables.nc59.

Research involving human participants, their data, or biological material

Policy information about studies with human participants or human data. See also policy information about sex, gender (identity/presentation), and sexual orientation and race, ethnicity and racism.

Reporting on sex and gender	NA
Reporting on race, ethnicity, or other socially relevant groupings	NA
Population characteristics	NA
Recruitment	NA
Ethics oversight	NA

Note that full information on the approval of the study protocol must also be provided in the manuscript.

Field-specific reporting

Please select the one below that is the best fit for your research. If you are not sure, read the appropriate sections before making your selection.

☐ Life sciences ☐ Behavioural & social sciences ☒ Ecological, evolutionary & environmental sciences

For a reference copy of the document with all sections, see nature.com/documents/nr-reporting-summary-flat.pdf

Ecological, evolutionary & environmental sciences study design

All studies must disclose on these points even when the disclosure is negative.

Study description	*Briefly describe the study. For quantitative data include treatment factors and interactions, design structure (e.g. factorial, nested, hierarchical), nature and number of experimental units and replicates.*
Research sample	NA
Sampling strategy	*Note the sampling procedure. Describe the statistical methods that were used to predetermine sample size OR if no sample-size calculation was performed, describe how sample sizes were chosen and provide a rationale for why these sample sizes are sufficient.*
Data collection	Satellite data
Timing and spatial scale	15 years from MLS and 7 years of SAGE-III dataset
Data exclusions	*If no data were excluded from the analyses, state so OR if data were excluded, describe the exclusions and the rationale behind them, indicating whether exclusion criteria were pre-established.*
Reproducibility	I used quality-controlled data provided by SAGE-III team.
Randomization	*Describe how samples/organisms/participants were allocated into groups. If allocation was not random, describe how covariates were controlled. If this is not relevant to your study, explain why.*

Blinding | *Describe the extent of blinding used during data acquisition and analysis. If blinding was not possible, describe why OR explain why blinding was not relevant to your study.*

Did the study involve field work? ☐ Yes ☒ No

Reporting for specific materials, systems and methods

We require information from authors about some types of materials, experimental systems and methods used in many studies. Here, indicate whether each material, system or method listed is relevant to your study. If you are not sure if a list item applies to your research, read the appropriate section before selecting a response.

Materials & experimental systems

n/a	Involved in the study
☒	☐ Antibodies
☒	☐ Eukaryotic cell lines
☒	☐ Palaeontology and archaeology
☒	☐ Animals and other organisms
☒	☐ Clinical data
☒	☐ Dual use research of concern
☒	☐ Plants

Methods

n/a	Involved in the study
☒	☐ ChIP-seq
☒	☐ Flow cytometry
☒	☐ MRI-based neuroimaging

Plants

Seed stocks	NA
Novel plant genotypes	NA
Authentication	NA
